# Intragraft transcriptional profiling of renal transplant patients with tubular dysfunction reveals mechanisms underlying graft injury and recovery

**DOI:** 10.1186/s40246-015-0059-6

**Published:** 2016-01-07

**Authors:** Hátylas Azevedo, Paulo Guilherme Renesto, Rogério Chinen, Erika Naka, Ana Cristina Carvalho de Matos, Marcos Antônio Cenedeze, Carlos Alberto Moreira-Filho, Niels Olsen Saraiva Câmara, Alvaro Pacheco-Silva

**Affiliations:** Department of Pediatrics, Faculdade de Medicina da Universidade de São Paulo (FMUSP), São Paulo, Brazil; Laboratory of Transplantation Immunobiology, Department of Immunology, Institute of Biomedical Sciences, Universidade de São Paulo (USP), São Paulo, Brazil; Laboratory of Clinical and Experimental Immunology, Nephrology Division, Universidade Federal de São Paulo (UNIFESP), São Paulo, Brazil; Instituto Israelita de Ensino e Pesquisa Albert Einstein, Hospital Albert Einstein, São Paulo, Brazil

**Keywords:** Network analysis, Kidney transplantation, Genomics, Proximal tubular dysfunction, Transcriptional profiling

## Abstract

**Background:**

Proximal tubular dysfunction (PTD) is associated with a decreased long-term graft survival in renal transplant patients and can be detected by the elevation of urinary tubular proteins. This study investigated transcriptional changes in biopsies from renal transplant patients with PTD to disclose molecular mechanisms underlying graft injury and functional recovery.

**Methods:**

Thirty-three renal transplant patients with high urinary levels of retinol-binding protein, a biomarker of PTD, were enrolled in the study. The initial immunosuppressive scheme included azathioprine, cyclosporine, and steroids. After randomization, 18 patients (group 2) had their treatment modified by reducing cyclosporine dosage and substituting azathioprine for mycophenolate mofetil, while the other 15 patients (group 1) remained under the initial scheme. Patients were biopsied at enrollment and after 12 months of follow-up, and paired comparisons were performed between their intragraft gene expression profiles. The differential transcriptome profiles were analyzed by constructing gene co-expression networks and identifying enriched functions and central nodes in each network.

**Results:**

Only the alternative immunosuppressive scheme used in group 2 ameliorated renal function and tubular proteinuria after 12 months of follow-up. Intragraft molecular changes observed in group 2 were linked to autophagy, extracellular matrix, and adaptive immunity. Conversely, gene expression changes in group 1 were related to fibrosis, endocytosis, ubiquitination, and endoplasmic reticulum stress.

**Conclusion:**

These results suggest that molecular networks associated with the control of endocytosis, autophagy, protein overload, fibrosis, and adaptive immunity may be involved in improvement of graft function.

**Electronic supplementary material:**

The online version of this article (doi:10.1186/s40246-015-0059-6) contains supplementary material, which is available to authorized users.

## Introduction

Proximal tubular dysfunction (PTD) is characterized by proteinuria, aminoaciduria, and glucosuria. It is associated with a decreased long-term graft survival in renal transplant patients and can be detected by the elevation of urinary tubular proteins [1, 2].

Low-molecular-weight proteins (LMWP) are prominent urine biomarkers of PTD [3–7], as they are cleared by glomerular filtration and almost totally reabsorbed by tubular epithelial cells. Specifically, increased urinary levels of the LMWP urinary retinol-binding protein (uRBP) have been associated with tubular injury and fibrosis after renal transplantation [8, 9]. Therefore, measuring uRBP levels may contribute to the detection of patients at a higher risk for renal function loss [10, 11].

The early detection of transplanted patients with kidney dysfunction helps optimizing their immunosuppression protocols in the attempt to improve graft outcomes. For instance, PTD is found in heart transplant patients with a progressive worsening on renal function due to cyclosporine (CsA) nephrotoxicity [12]. In parallel, immunosuppression with mycophenolate mofetil (MYF) is associated with less acute rejection after kidney transplantation [13], offering a therapeutic alternative for patients with renal dysfunction caused by CsA. However, a large number of transplant patients in developing countries still receive maintenance immunosuppression regimens containing low-cost drugs, including CsA, azathioprine (AZA), and steroids [14]. Hence, the follow-up of renal transplant patients diagnosed with PTD allows the adoption of tailored immunosuppression regimens and provides a window of opportunity for investigating renal injury and recovery.

Here, we followed up renal transplant patients with elevated uRBP levels that were submitted to specific immunosuppression regimens for 12 months. Kidney biopsies were performed to investigate intragraft transcriptional profiles and to correlate their molecular changes with renal function outcomes. Through this approach, we identified some potential molecular mechanisms associated with graft function improvement in renal transplant patients.

## Results

### Assessment of renal dysfunction in transplanted patients and association between tubular proteinuria and glomerular function rates

Table 1 summarizes the patients’ baseline clinical and laboratory features. None of these characteristics showed statistically significant differences (*p* < 0.05) between group 1 and group 2 patients. The analysis of renal histology in our cohort revealed no significant differences in the baseline Banff classification scores between the groups and also no significant differences in the Banff scores within the same group after 12 months of follow-up. At enrollment, the biopsies of three patients from group 1 showed no evidence of interstitial fibrosis and tubular atrophy (IF/TA), while nine, three, and zero biopsies showed mild, moderate, and severe IF/TA, respectively. At the end of the follow-up period, all biopsies from group 1 showed some evidence of IF/TA, with eight biopsies at mild, two at moderate, and two at severe IF/TA classification, respectively.

**Table 1:** Baseline clinical and laboratory characteristics of the renal transplant patients enrolled in this study (*n* = 33)

Variables	Group 1^a^ (*n* = 15)	Group 2^b^ (*n* = 18)	*p* value
Recipient age ± SD, in years	44.9 ± 11.2	45.4 ± 9.9	0.79
Recipient weight ± SD, in kg	67.5 ± 13.8	69.6 ± 14.7	0.35
Recipient male, *N* (%)	11 (73.3 %)	10 (55.6 %)	0.34
Deceased donors, *N* (%)	6 (40 %)	6 (33.3 %)	0.69
Haploidentical HLA, *N* (%)	5 (33.3 %)	10 (55.6 %)	0.20
Time post-transplant ± SD, in months	93.3 ± 35.7	92.4 ± 33.1	0.94
Systemic arterial hypertension, *N* (%)	13 (86.7 %)	16 (88.9 %)	0.84
Diabetes mellitus, *N* (%)	2 (13.3 %)	1 (5.6 %)	0.44
Serum creatinine levels ± SD, in mg/dL	1.59 ± 0.28	1.67 ± 0.33	0.46
uRBP levels ± SD, in mg/L	2.79 ± 2.55	2.72 ± 2.24	0.88
Banff classification^c^ at enrollment	No evidence (3), mild (9), moderate (3), severe (0)	No evidence (2), mild (9), moderate (5), severe (2)	0.64
Banff classification^c^ after 12 months	No evidence (0), mild (8), moderate (2), severe (2)	No evidence (3), mild (7), moderate (6), severe (1)	0.25

^a^Group 1 patients: initial immunosuppression scheme with cyclosporine, corticosteroids, and azathioprine

^b^Group 2 patients: alternative immunosuppression scheme with reduced levels of cyclosporine and use of mycophenolate mofetil

^c^Banff classification: mild, moderate, or severe evidence of interstitial fibrosis and tubular atrophy (IF/TA)

For group 2, the biopsies of two patients at enrollment showed no evidence of IF/TA, while nine, five, and two biopsies showed mild, moderate, and severe IF/TA, respectively. At the end of the follow-up period, three biopsies from group 2 showed no evidence of IF/TA, while the biopsies of seven, six, and one patients displayed mild, moderate, and severe IF/TA, respectively.

**Fig. 1:**
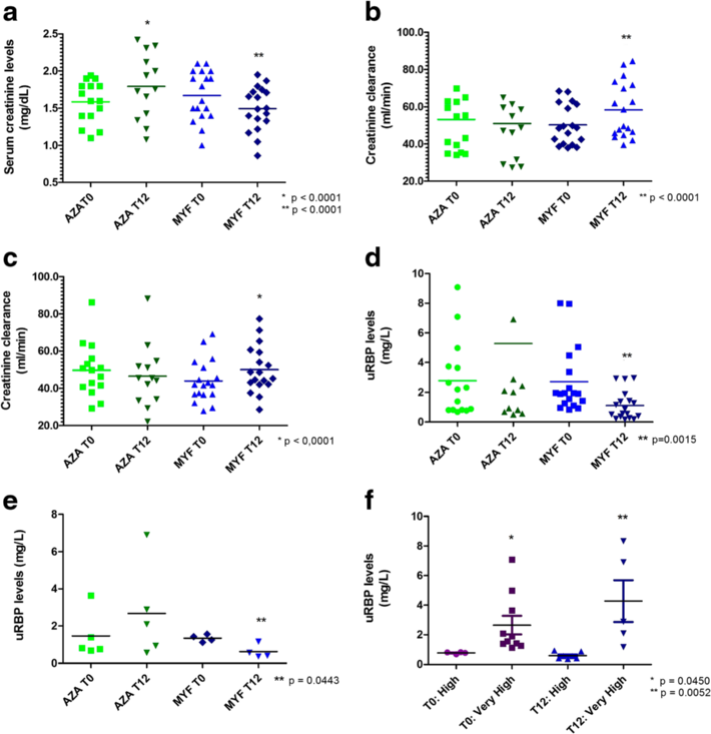
Measurement of serum creatinine, creatinine clearance, and urinary retinol-binding protein (*uRBP*) levels. **a** Serum creatinine levels (mg/dL) at the beginning and at the end of the follow-up period of 12 months. **b** Creatinine clearance (mL/min) calculated using Cockcroft-Gault formula. **c** Creatinine clearance (mL/min) calculated using MDRD formula. **d** uRBP levels (mg/L) from all enrolled patients. **e** uRBP levels (mg/L) from patients used for intragraft gene expression profiling. **f** Comparison between very high and high uRBP levels at t0 and t12 post-enrollment periods. Statistical significance was assessed using paired Student’s *t* test with *p* < 0.05. Renal function parameters were significantly altered in the groups treated with azathioprine (*AZA group comparison) or mycophenolate mofetil (**MYF group comparison) for 12 months

Figure 1 illustrates serum creatinine levels, estimated creatinine clearance, and uRBP levels at 0 and 12 months of follow-up. Interestingly, only patients from group 2 significantly improved these renal function parameters. Serum creatinine levels increased in group 1 after 12 months of follow-up, indicating a worsening in renal function (Fig. 1a). In parallel, creatinine clearance calculated by either Cockcroft-Gault (Fig. 1b) or modification of diet in renal disease (MDRD) (Fig. 1c) equations was only significantly improved in patients submitted to the alternative immunosuppression protocol (group 2). uRBP concentration was also decreased in patients from group 2 after 12 months (Fig. 1d, e).

**Fig. 2:**
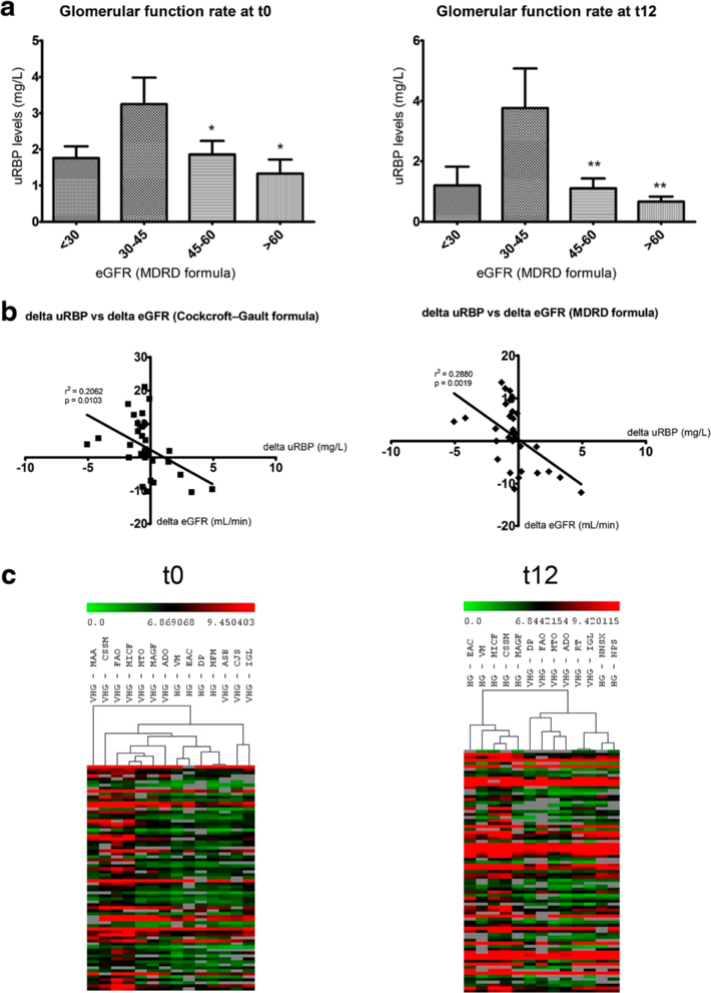
Distribution of uRBP levels according to glomerular filtration rates and hierarchical clustering analysis of samples from different ranges of uRBP levels. **a** Histograms showing uRBP values according to eGFR levels at enrollment (t0) and after 12 months of follow-up (t12). uRBP levels are expressed as the mean ± SEM. The estimated glomerular filtration rates (*eGFR*) were calculated using the MDRD formula. **p < 0.05, **p < 0.001 compared to eGFR at 30–45 mL/min, using ANOVA followed by Tukey’s correction. **b** Best-fit slope of the linear regression of the delta values from urinary retinol-binding protein (*uRBP*) levels and eGFR between t0 and t12. The estimated eGFR was calculated using Cockcroft-Gault or MDRD formulas. **c** Hierarchical clustering analysis of samples in the high group (*HG*, uRBP levels from 0.4 to 1 mg/L) and very high group (*VHG*, uRBP ≥ 1 mg/L) at t0 and t12

The distribution of uRBP values relative to estimated glomerular function rate (eGFR) equally showed a significant increase in RBP proteinuria in patients whose eGFR values ranged from 30 to 45 mL/min (with moderate kidney function reduction). uRBP levels in patients with eGFR <30 mL/min did not differ to those with eGFR values >45 mL/min (Fig. 2a).

A linear regression analysis was conducted to examine the association between delta values (t12–t0) for uRBP and eGFR over 12 months of follow-up. We found a small but significant correlation between delta values for uRBP and eGFR calculated via either Cockcroft-Gault (*r*^2^ = 0.2062, *p* = 0.0103) or MDRD (*r*^2^ = 0.2880, *p* = 0.0019) formulas, as depicted in Fig. 2b. This analysis showed that around 25 % of the variance in eGFR values was significantly explained by the variation in uRBP values.

### Network enrichment analysis reveals intragraft molecular changes associated with specific immunosuppression regimens

The lists of differentially expressed (DE) genes obtained in each comparison and their fold changes are displayed in Additional file 1. These DE genes were used to construct co-expression gene networks, and centrality measures were calculated for all nodes in order to determine the central nodes in each network. The centrality measures calculated were degree, which denotes the number of interactions a node has, and betweenness, which represents the fraction of shortest paths that passes through a specific node.

**Fig. 3:**
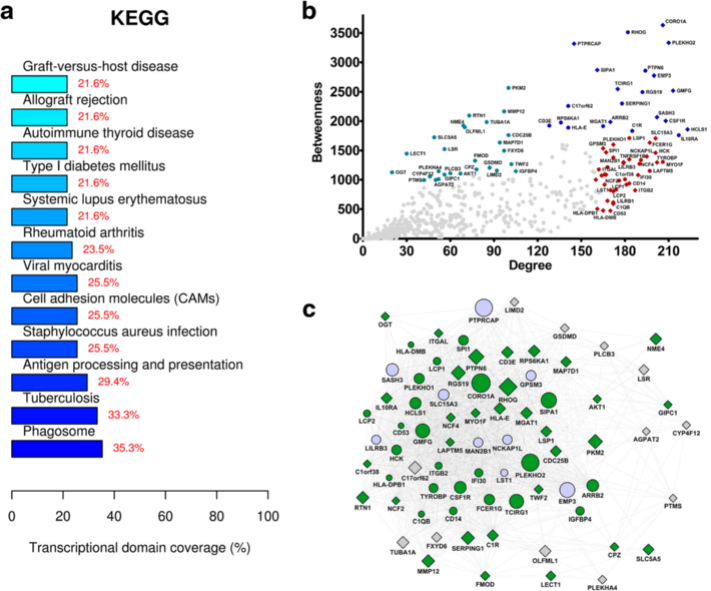
Analysis of the intragraft transcriptional network related to the alternative immunosuppression scheme (group 2 patients) after 12 months of follow-up. Network genes were obtained by performing paired comparisons between the intragraft transcriptional profiles (t12 versus t0) of patients submitted to the alternative immunosuppression scheme and by subsequently inputting those genes in the GeneMania tool. **a** KEGG categories showing enrichment in functions for the network nodes. **b** Scatterplot of degree versus betweenness centralities for nodes obtained in the transcriptional network analysis. High-hubs, hubs, and bottlenecks are depicted respectively in *royal blue*, *red*, and *oil blue colors*. **c** Transcriptional subnetwork containing the most central nodes in the group 2 network. Differentially expressed (DE) genes and DE-related genes are represented respectively as *gray diamonds* and *blue circles* in the network. Node sizes are based on betweenness centrality measurements. Genes previously associated with the keywords “autophagy,” “extracellular matrix,” “cell adhesion,” and “autoimmunity” are displayed in *green color*

**Fig. 4:**
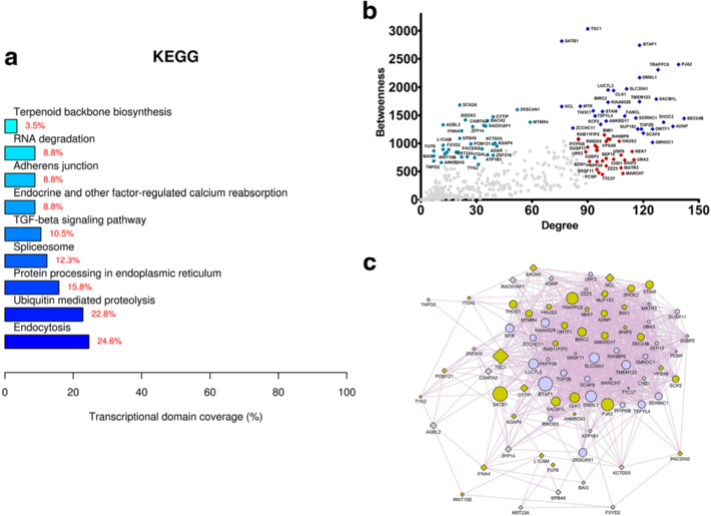
Analysis of the intragraft transcriptional network related to the standard immunosuppression scheme (group 1 patients) after 12 months of follow-up. Network genes were obtained by performing paired comparisons between the intragraft transcriptional profiles (t12 versus t0) of patients submitted to the standard immunosuppression scheme and by subsequently inputting those genes in the GeneMania tool. **a** KEGG categories showing enrichment in functions for the network nodes. **b** Scatterplot of degree versus betweenness centralities for nodes obtained in the transcriptional network analysis. High-hubs, hubs, and bottlenecks are depicted respectively in *royal blue*, *red*, and *oil blue colors*. **c** Transcriptional subnetwork containing the most central nodes in the group 1 network. Differentially expressed (DE) genes and DE-related genes are represented respectively as *gray diamonds* and *blue circles* in the network. Node sizes are based on betweenness centrality measurements. Genes previously associated with the keywords “endocytosis,” “fibrosis,” and “inflammation” are displayed in *beige color*

**Fig. 5:**
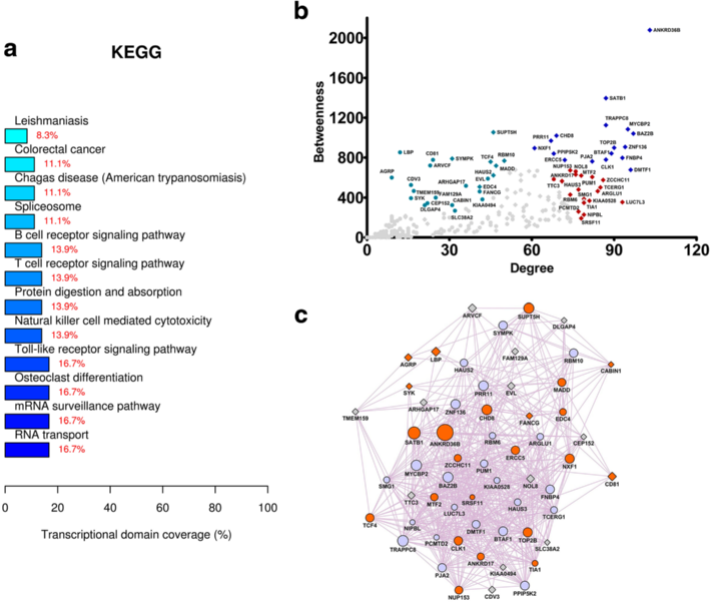
Analysis of the intragraft transcriptional network related to proximal tubular dysfunction (PTD) right after enrollment (t0). Network genes were obtained by comparing the intragraft transcriptional profiles of patients with elevated uRBP levels and by subsequently inputting those genes in the GeneMania tool. **a** KEGG categories showing enrichment in functions for the network nodes. **b** Scatterplot of degree versus betweenness centralities for nodes obtained in the transcriptional network analysis. High-hubs, hubs, and bottlenecks are depicted respectively in *royal blue*, *red*, and *oil blue colors*. **c** Transcriptional subnetwork containing the most central nodes in the t0 network. Differentially expressed (DE) genes and DE-related genes are represented respectively as *gray diamonds* and *blue circles* in the network. Node sizes are based on betweenness centrality measurements. Genes previously associated with the keywords “immune response,” “T regulatory cells,” “autophagy,” or “ubiquitin-proteasome” are displayed in *orange color*

**Fig. 6:**
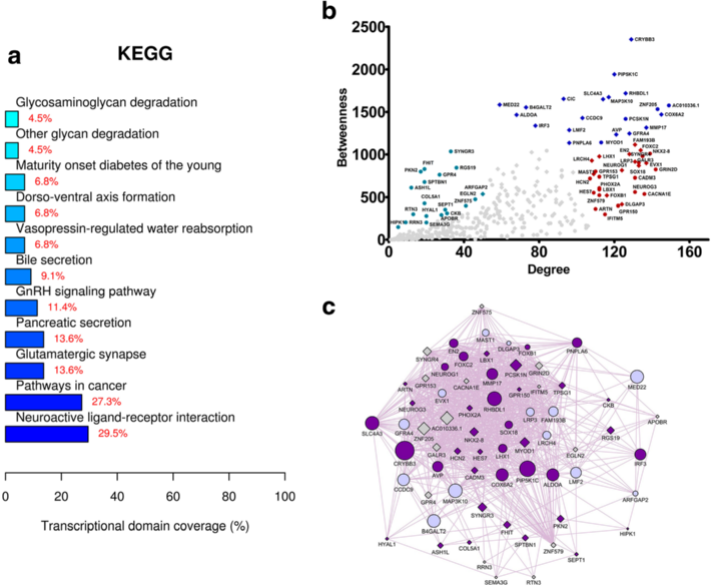
Analysis of the intragraft transcriptional network related to proximal tubular dysfunction (PTD) after 12 months of follow-up (t12). Network genes were obtained by comparing the intragraft transcriptional profiles of patients with elevated uRBP levels and by subsequently inputting those genes in the GeneMania tool. **a** KEGG categories showing enrichment in functions for the network nodes. **b** Scatterplot of degree versus betweenness centralities for nodes obtained in the transcriptional network analysis. High-hubs, hubs, and bottlenecks are depicted respectively in *royal blue*, *red*, and *oil blue colors*. **c** Transcriptional subnetwork containing the most central nodes in the t12 network. Differentially expressed (DE) genes and DE-related genes are represented respectively as *gray diamonds* and *blue circles* in the network. Node sizes are based on betweenness centrality measurements. Genes previously associated with the keywords “fibrosis” and “tissue repair” are displayed in *purple color*

These central nodes were then categorized in three subclasses of hubs, high-hubs, and bottlenecks, using the information from the scatterplots in Figs. 3b, 4b, 5b, and 6b. High-hubs, hubs, and bottlenecks are displayed in the scatterplots respectively at the up-right, down-right, and up-left quadrants, due to their differences in degree and betweenness values. This classification is in accordance with previous studies showing that node subclasses exert important functions in the context of health and disease [15–17]. For instance, hub genes regulate many other genes and tend to be essential in biological networks [18] and their node degrees increase in tumor networks, suggesting their association with gain of regulatory control [19]. In addition, high-hubs were linked to mechanisms underlying refractory epilepsy [20] and bottleneck genes were correlated with essentiality [21] in biological networks. The high-hubs, hubs, and bottlenecks are described in Additional file 2.

Enriched biological functions were revealed among central nodes in the subnetworks. These biological functions are displayed in Figs. 3a, 4a, 5a, and 6a. Biological functions enriched by the genes from group 1 and group 2 comparisons are shown respectively in Figs. 3 and 4. Genes derived from the group 1 comparison (group 1 network) were related to endocytosis, ubiquitin-mediated proteolysis, endoplasmic reticulum stress, TGF-β pathway, and adherens junctions. Conversely, genes from the group 2 comparison (group 2 network) were linked to phagosome, antigen processing and presentation, cell adhesion, autoimmunity, allograft rejection, and graft-versus-host disease. The subnetworks containing the most central nodes in each group are displayed in Figs. 3c to 6c. Genes associated with specific keywords in each case were marked in a different color, as described in the legend of each figure.

### Analysis of the intragraft molecular changes associated with higher retinol-binding proteinuria

We also associated the increased tubular proteinuria with a specific gene expression pattern, considering the differences in uRBP levels between the very high group (VHG) and high group (HG) (Fig. 1f). In addition, a hierarchical clustering analysis on the DE gene subsets was conducted to verify if the samples in each group showed similar gene expression patterns (Fig. 2c). With this approach, we confirmed that patients in the HG and VHG clustered together at t0 and t12, respectively.

Biological functions overrepresented by network genes from t0 and t12 comparisons are shown in Figs. 5 and 6. Genes derived from the former comparison (t0 network) are associated with osteoclast differentiation, protein digestion and absorption, and Toll-like, B cell, and T cell receptor pathways. On the other hand, genes derived from the latter comparison (t12 network) were linked to neuroactive ligand-receptor interaction, pathways in cancer, diabetes, and glycosaminoglycan degradation.

## Discussion

### Patients submitted to the alternative immunosuppression scheme show better renal function outcomes and a survival-related intragraft molecular profile

In the study cohort, patients that had their immunosuppression scheme altered by reducing the cyclosporine dosage and replacing AZA by MYF (group 2) improved serum creatinine and uRBP levels after 12 months of follow-up. Therefore, we hypothesized that distinct renal function outcomes could reflect intragraft molecular differences between patients from groups 1 and 2.

In the group 1 network (Fig. 3), the most overrepresented function was endocytosis. Therefore, endocytosis disturbance may be involved in proteinuria, as tubular injury is linked to the inhibition of LMWP receptor-mediated endocytosis [22]. Moreover, tubular atrophy biomarkers are associated with endocytosis in renal transplant patients [23]. Besides endocytosis, genes in the group 1 network were related to ubiquitination and endoplasmic reticulum stress. Endoplasmic reticulum stress is activated by unfolded protein accumulation and induces fibrosis in proteinuric kidney diseases [24, 25].

In the group 2 network (Fig. 4), the most overrepresented function was phagosome, a type of vesicle formed during phagocytosis and autophagy. Interestingly, autophagy plays a role in tissue homeostasis, as damaged structures are degraded by autolysosomes after injury [26, 27]. Moreover, the role of autophagy as a protective mechanism against ischemic injury was investigated in tubular cells [28] and autophagy activation by MYF was able to prolong cell survival [29]. Central nodes in the group 2 network were also related to autophagy, such as Coro1a, Akt1, Ifi30, Cd14, and Ncf4. Coro1a, for example, inhibits autophagosomes in macrophages [30].

Other overrepresented functions in the group 2 network were antigen presentation, autoimmune diseases, and allograft rejection. Two of these genes, Igfpb4 and Il10ra, were already associated with graft function loss [31]. Moreover, the up-regulated gene Klrb1 disclosed the existence of tolerance mechanisms related to FoxP3+ T cells [32]. Genes coding for class I and II major histocompatibility molecules (MHC) were also found in the group 2 network, highlighting the role of HLA-G (MHC class I) for protecting renal transplants from autoimmunity [33] and the function of Hla-dpb1 (MHC class II) in renal allograft rejection [34].

Finally, cell adhesion and extracellular matrix functions were overrepresented by genes in the group 2 network. Serping1 and Mmp12, for example, were already associated with parenchyma deterioration in allografts undergoing rejection [35, 36]. Therefore, the alternative immunosuppression regimen may exert part of its protective effects by regulating the extracellular matrix, considering that MYF reduces fibroblast infiltration, collagen deposition, and ECM synthesis in kidney [37].

### Patients with higher retinol-binding proteinuria have intragraft transcriptional changes associated with injury and repair mechanisms

Increased uRBP levels were significantly correlated with lower creatinine clearance in the cohort, corroborating the relevance of uRBP levels for assessing the extent of glomerular dysfunction after renal transplantation. Thus, we conducted a network analysis to identify molecular events correlated with higher tubular proteinuria.

Intragraft transcriptional changes in the t0 network (Fig. 5) reflected molecular mechanisms of immune-mediated allograft injury. Indeed, intragraft gene expression changes during transplant rejection are associated with immune response and precede the onset of IF/TA [38, 39]. In parallel, a gene subset in the t0 network was associated with the Foxp3+ T regulatory cell phenotype. The high-hub Satb1, for example, inhibits the transcription factor Foxp3 [40], and Srrm1, the most up-regulated gene in the t0 comparison (fold change = 23.9), is part of the Foxp3 network [41].

Central nodes in the t0 network such as Trappc8, Agrp, Syk, and Baz2b were also linked to autophagy and ubiquitin-proteasome functions. As a matter of fact, autophagy components interplay with ubiquitination processes [42], and autophagy exerts protective functions by inhibiting endoplasmic reticulum stress and ubiquitin-proteasome system [43]. Hence, the imbalance of these two protein degradation systems may be involved in kidney graft dysfunction.

In the t12 network, central nodes were associated with fibrosis (Fig. 6), like Irf3, Myod1, and Crybb3 [44–46]. Moreover, genes encoding actin and myosin, biomarkers of fibrosis, were highly up-regulated in the VHG (fold change around 300). Notably, the most overrepresented function in the t12 network was “neuroactive ligand-receptor interaction”, highlighting the role of neuronal genes for the response against ischemic renal injury [47] and maintenance of an epithelial phenotype in tubular cells [48].

## Conclusions

This study showed that increased retinol-binding proteinuria is associated with kidney function worsening in renal transplant patients. Patients under the alternative immunosuppressive scheme (group 2) improved renal function after 12 months of follow-up. The examination of their intragraft transcriptional profiles revealed mechanisms linked to graft injury and recovery. These results can be useful for further studying the mechanisms underlying graft injury and functional recovery. In particular, the role of genes participating in ubiquitination, autophagy, protein overload, and neuroactive ligand-receptor interaction should be further investigated.

## Methods

### Patients

We studied a cohort of 33 subjects who underwent renal transplantation in the Kidney Hospital at the Federal University of São Paulo. Patients with at least 1 year of transplant surgery were checked for uRBP levels during a 3-month follow-up. Patients with uRBP >0.4 mg/L in three consecutive monthly measurements were diagnosed with PTD, according to studies establishing this upper normal limit [2, 12], and sequentially included in this study. The study was approved by the Research Ethics Committee at UNIFESP (number 0420/05).

### Clinical study design

All patients enrolled were initially (t0) in use of CsA, prednisone, and AZA. They were randomized at enrollment for a prospective treatment with the standard protocol (group 1, *n* = 15) or with an alternative protocol with reduced CsA dosage and MYF introduction (group 2, *n* = 18). Patients were followed up for 12 months (t12) to determine if modifying their immunosuppression regimens could result in better renal function outcomes. All patients were submitted to graft biopsy at t0 and t12, and each biopsy was individually assessed in the transcriptomic profiling analysis.

### Kidney histological analysis

Histological findings were evaluated by a pathologist using optical microscopy. Biopsies were interpreted following the Banff classification for kidney allograft pathology: each biopsy was categorized according to classification grades of interstitial fibrosis and tubular atrophy (IF/TA), which establishes the degree (I, II, and III) of interstitial fibrosis (mild, moderate, severe) and tubular atrophy (mild to moderate) for each evaluated sample [49]. Histological differences between the groups were determined using the *χ*^2^ test for the categorical variables.

### Renal function evaluation

Serum creatinine, creatinine clearance, and uRBP levels were measured in patients at enrollment and at the end of the follow-up period. Serum creatinine and uRBP levels were measured according to described elsewhere [10]. The glomerular function rate (eGFR) estimation was based on serum creatinine concentration and calculated using the Cockcroft-Gault and the modification of diet in renal disease (MDRD) formulas. The prediction of creatinine clearance by the Cockcroft-Gault equation was calculated as (140 − age) × body weight/plasma creatinine × 72 (×0.85 if female), and the MDRD estimate was determined as 175 × plasma creatinine − 1.154 × age − 0.203 (×0.742 if female; ×1.21 if black). Serum creatinine levels are represented in mg/dL, creatinine clearance in mL/min, and uRBP levels in mg/L. Statistical analysis (Student’s *t* tests, with *p* < 0.05) was performed using GraphPad Prism 5.

### Comparative analysis of the intragraft gene expression profiles

To investigate transcriptional changes related to specific immunosuppressive regimens, we performed paired comparisons (t12 versus t0) between biopsies from the same patients in group 1 (group 1 comparison) and in group 2 (group 2 comparison). A second analysis was performed to determine transcriptional changes associated with increased retinol-binding proteinuria. Patients were divided into subgroups: patients with uRBP values above 1.0 mg/L were classified in the very high group (VHG), whereas patients with uRBP levels above 0.4 mg/L but less than 1.0 mg/L were classified in the high group (HG). The uRBP cutoff level of 1.0 mg/L was selected because renal transplant patients with uRBP levels ≥1 mg/L are at a much higher risk for kidney function deterioration [10]. Moreover, the average uRBP levels in transplant patients with an estimated GFR less than 60 mL/min (i.e., with moderate to severe renal damage) were also higher than 1 mg/L [12]. The comparative analyses were done between the transcriptional profiles of patients from the VHG and HG at t0 (t0 comparison) and t12 (t12 comparison).

### Oligonucleotide microarray experiments

Microarray experiments were performed as previously described [50]. Only samples with a RNA integrity number greater than 7 were employed in microarray experiments. Gene expression profiles were assessed using Agilent whole human genome 4x44K oligonucleotide microarrays. The R environment (http://www.r-project.org) was used to filter and pre-process the data. The mean of the probes for each gene was calculated, and the signal intensities were log2 transformed. These logarithmic values were input in the TmeV software to perform the statistical analyses [51]. No further normalization step was performed after visual inspection of the data distribution across the samples. The complete data set is available at the National Center for Biotechnology Information (NCBI) Gene Expression Omnibus, through the accession number GSE48250.

### Bioinformatics workflow

The analyses described below were performed to obtain further information on the comparative gene expression profiles.

i) *Statistical testing.* Differentially expressed (DE) genes were found by paired (group 1 and group 2 comparisons) and unpaired (VHG × HG comparisons) *t* tests, with *p* < 0.05 as the significance threshold. The false discovery rate (FDR) control was initially applied to adjust the *p* values for multiple comparisons, but only few significant genes were obtained using this procedure. Therefore, a less stringent approach was used by analyzing the data with no FDR adjustment, with the understanding that false positives were not restrained a priori. In addition, the FDR corrections were not used in order to obtain a relevant number of genes for the network enrichment analysis.
ii) *Hierarchical clustering analysis.* Hierarchical clustering (HCL) analysis [52] was performed using TmeV. HCL was used to group DE genes based on their expression similarities across the samples. The average distance clustering method was employed, using sample tree selection and sample leaf order optimization. The distance metric used was the Pearson correlation, and HCL was performed only in the significant genes to reduce cluster noise.
iii) *Gene co-expression network analysis.* The differential transcriptomic datasets were used to generate the gene co-expression networks. The Cytoscape plug-in GeneMANIA [53] was used to predict DE gene interactions. Networks were generated using information from the co-expression category in GeneMANIA. Genes that co-express with DE genes (DE-related genes) were also included in the networks to study the interactions between DE genes and other co-expressed genes. To analyze the centrality of the nodes (genes) contained in the networks, the node centrality parameters “degree” and “betweenness” were calculated using the Cytoscape plug-in CentiScaPe [54]. Node degree is a local structure measure in networks that determines the number of edges linked to a node. Conversely, betweenness centrality is a global structure measure that defines the fraction of shortest paths passing through a node.
iv) *Identification of high-hubs, hubs, and bottlenecks.* Scatterplots were constructed using degree and betweenness values for each node in GraphPad Prism®5. These scatterplots allowed node hierarchy categorization in high-hubs, hubs, and bottlenecks. This categorization takes in account gene localization in different quadrants of the graph. High-hubs are placed in the up-right quadrant due to their higher degree and betweenness values. Conversely, hubs are located in the down-right quadrant, as they present high degree but lower betweenness values compared to high-hubs. Finally, bottlenecks are located in the up-left quadrant, as they show high betweenness but low degree values.
v) *Functional enrichment analysis*. Overrepresented biological functions were searched in the differential transcriptomic datasets using FunNet, a bioinformatics tool that performs functional profiling of gene expression data [55].
vi) *Analysis of subnetworks derived from central nodes.* Subnetworks were built with the aid of Cytoscape [56], using the central nodes identified in each comparison. Semantic relationships were identified between genes and keywords with the text mining tool GenClip [57]. We searched for relationships using the keywords “immune response”, “T regulatory cells”, “autophagy”, “ubiquitin-proteasome”, “endocytosis”, “fibrosis”, “inflammation”, “extracellular matrix”, “cell adhesion”, and “autoimmunity”. These relationships were highlighted in each subnetwork.

## Additional files

Additional file 1:
**Differentially expressed genes obtained in each comparison.** This table contains the differentially expressed (DE) genes obtained in each comparison and their fold changes. Statistical analysis using unpaired and paired *t* tests identified 250, 434, 417, and 593 DE genes according to the respective comparisons: t0 (very high versus high uRBP levels), t12 (very high versus high uRBP levels), group 1 (t12 × t0), and group 2 (t12 × t0). Additional file 2:
**High-hub, hub, and bottleneck genes identified in each comparison.** Hubs were defined as highly connected nodes according to node degree values. High-hubs are top-ranked hubs presenting also high betweenness centrality values. Bottleneck genes were classified as nodes with high betweenness centrality but low node degree values.

## Abbreviations

AZA: azathioprine
CsA: cyclosporine
DE: differentially expressed
eGFR: estimated glomerular function rate
HCL: hierarchical clustering
HG: high group
IF/TA: interstitial fibrosis and tubular atrophy
LMWP: low-molecular-weight proteins
MDRD: modification of diet in renal diseases
MYF: mycophenolate mofetil
PTD: proximal tubular dysfunction
uRBP: urinary retinol-binding protein
VHG: very high group
