# Supplementary material for: Intragraft transcriptional profiling of renal transplant patients with tubular dysfunction reveals mechanisms underlying graft injury and recovery
Source: Hum Genomics. 2016 Jan 7;10:2. doi: 10.1186/s40246-015-0059-6 (PMC4705764; doi:10.1186/s40246-015-0059-6)
Supplement: Additional file 1: — Differentially expressed genes obtained in each comparison. This table contains the differentially expressed (DE) genes obtained in each comparison and their fold changes. Statistical analysis using unpaired and paired t tests identified 250, 434, 417, and 593 DE genes according to the respective comparisons: t0 (very high versus high uRBP levels), t12 (very high versus high uRBP levels), group 1 (t12 × t0), and group 2 (t12 × t0). [file 40246_2015_59_MOESM1_ESM.pdf]

Group 1 Comparison				
**ProbeName**	**SystematicName**	**GeneName**	**p value**	**Fold (t12 / t0)**
A_32_P179295	BC020462	RPS9	0.047	2.334
A_32_P111658	N91552	N91552	0.035	2.145
A_32_P212685	BX091616	BX091616	0.045	2.012
A_32_P48256	DB381305	DB381305	0.033	1.984
A_24_P400573	NM_152468	TMC8	0.002	1.917
A_32_P157415	BE714413	BE714413	0.046	1.874
A_32_P27327	W27166	W27166	0.033	1.862
A_23_P43380	NM_025182	KIAA1539	0.046	1.828
A_32_P43914	THC2443137	THC2443137	0.031	1.792
A_23_P153964	NM_002193	INHBB	0.048	1.778
A_24_P102119	AF264623	AF264623	0.026	1.752
A_23_P300087	NM_032030	FKSG83	0.006	1.749
A_24_P708161	A_24_P708161	A_24_P708161	0.005	1.690
A_23_P43977	A_23_P43977	A_23_P43977	0.040	1.686
A_23_P104741	ENST00000278934	ENST00000278934	0.043	1.663
A_23_P136296	A_23_P136296	A_23_P136296	0.039	1.656
A_24_P759955	THC2280373	THC2280373	0.025	1.594
A_32_P187919	AF086156	AF086156	0.012	1.587
A_23_P45185	NM_004469	FIGF	0.021	1.576
A_23_P116624	NM_004211	SLC6A5	0.030	1.576
A_23_P30634	NM_021813	BACH2	0.018	1.573
A_24_P229871	NM_182564	C17orf54	0.007	1.550
A_32_P163014	AI580662	AI580662	0.013	1.507
A_32_P70372	BX448200	BX448200	0.039	1.504
A_23_P93524	NM_001017373	SAMD3	0.024	1.487
A_23_P206806	NM_002209	ITGAL	0.015	1.469
A_23_P310956	NM_058175	COL6A2	0.032	1.465
A_23_P86874	NM_033347	KCNK7	0.008	1.449
A_23_P74701	NM_152890	COL24A1	0.028	1.434
A_24_P918441	AK096056	AK096056	0.048	1.427
A_32_P207231	AI630435	AI630435	0.027	1.426
A_32_P8857	A_32_P8857	A_32_P8857	0.048	1.424
A_23_P378514	NM_145178	ATOH7	0.039	1.423
A_32_P481377	NM_175858	KRTAP11-1	0.049	1.417
A_32_P178746	THC2385388	THC2385388	0.024	1.411
A_32_P28634	THC2366887	THC2366887	0.031	1.402
A_23_P252556	NM_001704	BAI3	0.016	1.401
A_23_P171270	NM_001017991	H2AFB2	0.014	1.397
A_24_P230521	CR623296	GPSM3	0.034	1.387
A_24_P916031	AL138396	AL138396	0.004	1.371
A_24_P790909	AI652176	AI652176	0.047	1.371
A_32_P171790	AF113008	AF113008	0.037	1.369
A_23_P51019	NM_021007	SCN2A2	0.035	1.368
A_32_P200025	A_32_P200025	A_32_P200025	0.039	1.362
A_24_P337867	NM_032831	TMEM142B	0.046	1.347
A_24_P383478	NM_000125	ESR1	0.019	1.333
A_23_P202245	ENST00000355710	ENST00000355710	0.049	1.330
A_23_P9472	NM_033305	VPS13A	0.034	1.314

A_23_P127697	ENST00000331596	ENST00000331596	0.023	1.312
A_24_P255609	ENST00000324745	ENST00000324745	0.037	1.307
A_32_P3914	THC2437430	THC2437430	0.005	1.304
A_32_P125416	BX115105	BX115105	0.018	1.300
A_24_P24263	NM_001011515	PDLIM5	0.042	1.299
A_32_P200970	BM668321	BM668321	0.036	1.293
A_23_P356070	NM_002020	FLT4	0.046	1.275
A_32_P32254	NM_001848	COL6A1	0.027	1.257
A_23_P162322	NM_003394	WNT10B	0.008	1.250
A_32_P116105	BC024649	BC024649	0.034	1.244
A_23_P9485	NM_000608	ORM2	0.035	1.232
A_24_P349274	NM_001004727	OR4X2	0.019	1.224
A_32_P450879	THC2402213	THC2402213	0.035	1.214
A_23_P16089	NM_004230	EDG5	0.026	1.205
A_24_P403459	NM_021068	IFNA4	0.022	1.202
A_32_P11701	BF939434	BF939434	0.028	1.199
A_24_P102389	NM_001004127	ALG11	0.005	1.195
A_24_P936376	ENST00000367058	ENST00000367058	0.012	1.192
A_23_P148602	NM_003669	INE1	0.023	1.184
A_23_P137634	NM_002763	PROX1	0.033	1.179
A_23_P153741	NM_001700	AZU1	0.036	1.170
A_24_P74896	NM_182611	GPR144	0.015	1.156
A_24_P320699	NM_001013398	IGFBP3	0.024	1.145
A_32_P37733	AK098081	AK098081	0.034	1.136
A_32_P182502	BC044608	BC044608	0.041	1.134
A_23_P369574	NM_015097	CLASP2	0.039	1.129
A_32_P22989	A_32_P22989	A_32_P22989	0.013	1.116
A_23_P361841	ENST00000357529	ENST00000357529	0.042	1.114
A_23_P430670	NM_024533	CHST5	0.036	1.110
A_23_P82286	NM_173059	ZAN	0.047	1.106
A_32_P221569	BM989272	BM989272	0.022	1.076
A_24_P151295	NM_005934	MLLT1	0.032	1.074
A_23_P130836	NM_005317	GZMM	0.039	1.059
A_23_P355623	AL137349	ANKIB1	0.045	1.039
A_24_P361408	NM_173623	TTLL6	0.046	1.038
A_23_P88781	NM_020313	CIAPIN1	0.015	1.030
A_32_P94801	THC2308876	THC2308876	0.021	1.026
A_23_P351069	NM_003955	SOCS3	0.036	1.024
A_32_P188263	AW383740	AW383740	0.013	1.019
A_23_P47682	NM_020645	NRIP3	0.026	1.015
A_23_P159119	NM_016507	CRKRS	0.049	1.014
A_23_P350574	NM_001002901	FCRLM2	0.017	1.009
A_23_P80449	NM_000461	THRB	0.024	1.006
A_23_P123943	NM_004612	TGFBR1	0.042	-1.002
A_23_P106835	NM_031885	BBS2	0.035	-1.004
A_23_P6293	NM_018961	UBASH3A	0.002	-1.005
A_23_P13797	NM_024738	C12orf49	0.038	-1.028
A_24_P261203	ENST00000372146	ENST00000372146	0.031	-1.035
A_24_P346431	NM_022748	TNS3	0.029	-1.037
A_23_P213562	NM_001992	F2R	0.003	-1.049

A_24_P932418	NM_012305	AP2A2	0.037	-1.052
A_23_P143987	NM_006395	ATG7	0.048	-1.053
A_23_P41365	NM_012390	SMR3A	0.025	-1.055
A_23_P83234	NM_006626	ZBTB6	0.042	-1.056
A_24_P158089	NM_000602	SERPINE1	0.039	-1.059
A_24_P169937	NM_178844	NOD3	0.043	-1.064
A_32_P116857	AK127194	AK127194	0.038	-1.068
A_23_P207719	NM_004287	GOSR2	0.004	-1.070
A_24_P938006	A_24_P938006	A_24_P938006	0.024	-1.071
A_24_P490911	AK023756	AK023756	0.040	-1.079
A_24_P941148	NM_017645	FAM29A	0.045	-1.079
A_24_P538708	AK124263	AK124263	0.048	-1.080
A_32_P34522	BC010544	BC010544	0.011	-1.083
A_24_P119519	NM_002311	LIG3	0.018	-1.085
A_32_P157775	THC2375853	THC2375853	0.034	-1.089
A_24_P282363	NM_032529	KIAA1875	0.042	-1.089
A_24_P199500	NM_007212	RNF2	0.003	-1.092
A_32_P7176	AW949170	AW949170	0.043	-1.102
A_32_P34046	NM_198550	SEC63D1	0.012	-1.109
A_23_P29384	NM_033210	ZNF502	0.037	-1.111
A_24_P944502	A_24_P944502	A_24_P944502	0.004	-1.115
A_23_P20676	NM_024635	MAK10	0.009	-1.120
A_23_P48535	NM_001173	ARHGAP5	0.041	-1.123
A_23_P377267	AB007940	RABGAP1L	0.019	-1.125
A_23_P208172	AK023652	ZNF613	0.039	-1.127
A_23_P252664	U27333	FUT6	0.009	-1.128
A_23_P325411	ENST00000379938	ENST00000379938	0.033	-1.129
A_23_P166159	NM_030815	PDRG1	0.032	-1.131
A_23_P124742	NM_001277	CHKA	0.046	-1.136
A_23_P101427	NM_145245	EVI5L	0.002	-1.137
A_24_P199097	NM_006809	TOMM34	0.021	-1.140
A_23_P313640	NM_006609	MAP3K2	0.036	-1.143
A_23_P128319	NM_001682	ATP2B1	0.011	-1.147
A_23_P125772	NM_014370	STK23	0.027	-1.148
A_23_P170839	NM_001008491	2-Sep	0.023	-1.149
A_23_P4604	NM_198457	ZNF600	0.005	-1.160
A_23_P56798	NM_004300	ACP1	0.048	-1.162
A_23_P386450	NM_016218	POLK	0.013	-1.165
A_24_P252705	NM_004412	DNMT2	0.036	-1.173
A_23_P84872	NM_024077	SECISBP2	0.041	-1.175
A_23_P98532	NM_015457	ZDHHC5	0.031	-1.177
A_24_P186342	NM_020917	ZFP14	0.043	-1.178
A_23_P90626	NM_004288	PSCDBP	0.019	-1.179
A_23_P106016	NM_002742	PRKD1	0.046	-1.179
A_23_P374351	NM_173562	KCTD20	0.045	-1.182
A_24_P183292	NM_001025105	CSNK1A1	0.049	-1.182
A_32_P222857	NM_002139	RBMX	0.003	-1.188
A_32_P86009	A_32_P86009	A_32_P86009	0.003	-1.189
A_24_P144149	A_24_P144149	A_24_P144149	0.042	-1.190
A_23_P55518	NM_005904	SMAD7	0.018	-1.197

A_32_P210723	THC2448556	THC2448556	0.037	-1.199
A_23_P133474	NM_002084	GPX3	0.029	-1.201
A_23_P122906	NM_015570	AUTS2	0.037	-1.202
A_23_P154297	NM_032144	RAB6C	0.040	-1.221
A_24_P106910	ENST00000375284	ENST00000375284	0.028	-1.223
A_23_P203009	NM_003478	CUL5	0.041	-1.223
A_23_P154488	NM_033109	PNPT1	0.022	-1.224
A_23_P378499	AK095607	FLJ38288	0.021	-1.229
A_24_P254285	NM_052865	C20orf72	0.042	-1.229
A_23_P216402	NM_031432	UCK1	0.012	-1.230
A_23_P32217	NM_005802	TOPORS	0.008	-1.230
A_24_P90878	NM_017736	THUMPD1	0.037	-1.233
A_24_P349648	A_24_P349648	A_24_P349648	0.021	-1.234
A_24_P329635	NM_000368	TSC1	0.020	-1.245
A_23_P36305	NM_033388	ATG16L2	0.038	-1.248
A_24_P349580	A_24_P349580	A_24_P349580	0.001	-1.250
A_23_P80377	NM_007229	PACSIN2	0.009	-1.251
A_23_P86822	NM_014679	CEP57	0.012	-1.252
A_32_P13442	A_32_P13442	A_32_P13442	0.005	-1.254
A_23_P112883	NM_178564	NRBP2	0.049	-1.259
A_24_P317637	NM_014569	ZFP95	0.047	-1.259
A_32_P38003	ENST00000379156	ENST00000379156	0.027	-1.260
A_23_P67708	NM_003200	TCF3	0.042	-1.261
A_23_P80891	NM_003363	USP4	0.041	-1.262
A_23_P207620	NM_016376	ANKFY1	0.004	-1.263
A_23_P68155	NM_022168	IFIH1	0.019	-1.264
A_32_P82119	A_32_P82119	A_32_P82119	0.041	-1.265
A_23_P161297	NM_018245	OGDHL	0.024	-1.266
A_24_P62615	NM_006367	CAP1	0.044	-1.267
A_23_P132595	NM_014667	VGLL4	0.036	-1.267
A_32_P23960	AI650707	AI650707	0.048	-1.268
A_23_P416894	AK126364	LOC54103	0.044	-1.270
A_23_P20876	NM_177995	PTPDC1	0.032	-1.274
A_23_P38365	NM_006852	TLK2	0.021	-1.275
A_23_P170290	NM_018202	TMEM57	0.028	-1.276
A_23_P386764	NM_020438	DOLPP1	0.037	-1.277
A_23_P54064	NM_019852	METTL3	0.045	-1.277
A_24_P910325	XM_496278	ZNF516	0.027	-1.279
A_23_P55123	NM_001303	COX10	0.049	-1.284
A_24_P940499	D80006	D80006	0.023	-1.291
A_23_P161769	NM_021603	FXYD2	0.037	-1.292
A_23_P16354	NM_032825	ZNF382	0.030	-1.292
A_32_P190316	THC2377297	THC2377297	0.039	-1.292
A_23_P395566	NM_024735	FBXO31	0.026	-1.297
A_23_P164637	NM_024691	ZNF419	0.008	-1.298
A_23_P354953	NM_013433	TNPO2	0.035	-1.299
A_24_P409670	AK098175	ZNF283	0.018	-1.302
A_24_P662177	THC2448843	THC2448843	0.022	-1.302
A_32_P49211	AK055620	LOC286170	0.039	-1.302
A_23_P166135	NM_012255	XRN2	0.029	-1.302

A_23_P106	NM_015017	USP33	0.037	-1.302
A_23_P400406	NM_175873	ANKRD43	0.034	-1.303
A_23_P210445	NM_015478	L3MBTL	0.030	-1.303
A_23_P164237	NM_018428	UTP6	0.029	-1.310
A_24_P205458	NM_003607	CDC42BPA	0.002	-1.314
A_24_P174641	NM_006457	PDLIM5	0.037	-1.316
A_23_P414895	NM_018259	TTC17	0.021	-1.319
A_24_P167052	NM_139312	YME1L1	0.050	-1.322
A_24_P332364	A_24_P332364	A_24_P332364	0.032	-1.323
A_24_P822692	XM_933624	LOC643669	0.045	-1.324
A_24_P177553	THC2267302	THC2267302	0.004	-1.325
A_23_P99292	NM_006479	RAD51AP1	0.040	-1.325
A_24_P4661	NM_004879	EI24	0.031	-1.326
A_32_P137632	BC018548	FBXL17	0.022	-1.326
A_32_P230196	NM_133446	CTGLF1	0.034	-1.329
A_32_P58464	NR_002228	LOC152667	0.046	-1.330
A_24_P764598	NM_080392	PTP4A2	0.043	-1.331
A_24_P351304	NM_006839	IMMT	0.017	-1.333
A_24_P136551	AB040932	NPLOC4	0.006	-1.334
A_24_P407704	THC2434479	THC2434479	0.024	-1.335
A_24_P15877	AF333762	AF333762	0.028	-1.335
A_23_P133315	NM_003432	ZNF131	0.046	-1.338
A_24_P319369	NM_144503	F11R	0.044	-1.338
A_23_P396981	NM_001012506	CCDC66	0.037	-1.339
A_24_P915149	AA916168	AA916168	0.027	-1.339
A_23_P15857	NM_005134	PPP4R1	0.016	-1.342
A_23_P160828	NM_017891	C1orf159	0.010	-1.343
A_23_P19095	NM_014035	SNX24	0.027	-1.345
A_23_P142380	NM_005858	AKAP8	0.027	-1.345
A_23_P204380	NM_024312	GNPTAB	0.039	-1.345
A_23_P3775	NM_018233	OGFOD1	0.048	-1.347
A_24_P319942	NM_007107	SSR3	0.023	-1.348
A_23_P410982	THC2430400	THC2430400	0.024	-1.348
A_23_P28538	NM_031902	MRPS5	0.001	-1.349
A_23_P140050	NM_001014286	FAM48A	0.013	-1.350
A_24_P383751	A_24_P383751	A_24_P383751	0.037	-1.354
A_24_P362317	NM_001111	ADAR	0.003	-1.354
A_23_P203933	NM_002223	ITPR2	0.045	-1.355
A_24_P273245	A_24_P273245	A_24_P273245	0.013	-1.358
A_23_P59481	NM_014671	UBE3C	0.013	-1.359
A_23_P5551	NM_005381	NCL	0.012	-1.359
A_32_P75661	THC2309312	THC2309312	0.047	-1.361
A_32_P167705	NM_024783	AGBL2	0.037	-1.361
A_23_P104065	NM_012405	ICMT	0.016	-1.363
A_23_P21230	NM_015294	TRIM37	0.039	-1.363
A_23_P321160	AB058774	ZNF594	0.040	-1.365
A_32_P53884	ENST00000354271	ENST00000354271	0.007	-1.366
A_23_P92320	NM_017426	NUP54	0.044	-1.367
A_23_P160460	NM_003115	UAP1	0.048	-1.369
A_23_P2801	NM_172373	ELF1	0.002	-1.370

A_32_P179837	NM_174903	RNF151	0.014	-1.373
A_23_P80129	NM_003683	D21S2056E	0.039	-1.373
A_24_P76319	A_24_P76319	A_24_P76319	0.024	-1.373
A_23_P31389	NM_013293	TRA2A	0.040	-1.373
A_24_P417281	NM_019022	TXNDC10	0.039	-1.378
A_23_P110090	AK002004	WDR52	0.037	-1.379
A_24_P400970	A_24_P400970	A_24_P400970	0.023	-1.379
A_23_P417942	NM_001024948	FNBP1L	0.040	-1.380
A_24_P218587	NM_004268	CRSP6	0.033	-1.380
A_24_P267997	NM_015602	TOR1AIP1	0.033	-1.381
A_23_P68966	CR626044	CR626044	0.021	-1.383
A_24_P321184	A_24_P321184	A_24_P321184	0.011	-1.384
A_23_P42241	NM_030876	OR5V1	0.046	-1.384
A_23_P500251	NM_003404	YWHAB	0.003	-1.385
A_32_P187458	BQ233242	BQ233242	0.047	-1.387
A_23_P110624	NM_001332	CTNND2	0.020	-1.388
A_24_P919920	AK092987	ARV1	0.002	-1.389
A_23_P209879	AK128731	ATF2	0.006	-1.391
A_23_P370588	NM_024016	HOXB8	0.038	-1.394
A_24_P655646	AL137495	AL137495	0.029	-1.394
A_23_P211973	NM_024800	NEK11	0.037	-1.400
A_23_P94319	NM_014867	KBTBD11	0.041	-1.401
A_23_P207879	NM_024110	CARD14	0.020	-1.405
A_23_P2692	NM_031431	COG3	0.022	-1.406
A_23_P387552	NM_000966	RARG	0.034	-1.406
A_32_P35452	THC2279497	THC2279497	0.027	-1.411
A_23_P314115	NM_005180	BMI1	0.045	-1.411
A_23_P3856	NM_153688	ZFP1	0.042	-1.411
A_23_P202484	NM_032772	ZNF503	0.018	-1.412
A_23_P328652	NM_153240	NPHP3	0.016	-1.412
A_24_P530900	A_24_P530900	A_24_P530900	0.050	-1.412
A_32_P199217	THC2458577	THC2458577	0.050	-1.413
A_24_P313334	AK094846	AK094846	0.035	-1.416
A_24_P1167	NM_017987	RUFY2	0.046	-1.419
A_23_P117971	M15530	M15530	0.036	-1.422
A_24_P63118	NM_013374	PDCD6IP	0.020	-1.423
A_23_P323094	NM_004426	PHC1	0.047	-1.424
A_23_P44643	NM_016238	ANAPC7	0.026	-1.425
A_23_P105307	NM_201444	DGKA	0.049	-1.426
A_24_P390833	NM_023075	MPPE1	0.023	-1.426
A_23_P205355	NM_000624	SERPINA5	0.035	-1.431
A_24_P570583	NM_194319	ZNF542	0.042	-1.437
A_23_P165582	NM_006226	PLCL1	0.024	-1.438
A_23_P149812	NM_012425	RSU1	0.039	-1.439
A_32_P98291	ENST00000361972	ENST00000361972	0.008	-1.441
A_24_P592591	THC2270231	THC2270231	0.046	-1.442
A_32_P7193	A_32_P7193	A_32_P7193	0.000	-1.444
A_24_P394368	NM_025132	WDR19	0.020	-1.447
A_23_P50418	NM_153358	FLJ90396	0.013	-1.448
A_24_P117029	NM_000527	LDLR	0.001	-1.452

A_23_P125541	NM_033215	PPP1R3F	0.025	-1.458
A_24_P594094	A_24_P594094	A_24_P594094	0.032	-1.459
A_23_P140057	NM_018647	TNFRSF19	0.004	-1.463
A_24_P500621	AK074291	AK074291	0.031	-1.463
A_23_P50156	NM_004869	VPS4B	0.040	-1.465
A_23_P135015	BC001725	KIAA0258	0.038	-1.468
A_23_P219117	NM_001978	EPB49	0.043	-1.475
A_24_P901986	BC029255	BC029255	0.036	-1.479
A_23_P116829	NM_003348	UBE2N	0.032	-1.480
A_24_P73669	NM_002094	GSPT1	0.017	-1.481
A_24_P380348	NM_021940	SMAP1	0.027	-1.482
A_24_P219920	NM_018708	FEM1A	0.022	-1.483
A_24_P370887	NM_004781	VAMP3	0.034	-1.483
A_32_P42913	AI051172	AI051172	0.048	-1.485
A_24_P32473	NM_024930	ELOVL7	0.036	-1.486
A_23_P218717	NM_014948	UBOX5	0.021	-1.490
A_24_P14776	BX647688	SYTL5	0.021	-1.492
A_24_P176131	NM_052950	WDFY2	0.028	-1.495
A_23_P365189	NM_015099	CAMTA2	0.018	-1.498
A_23_P202769	NM_005528	DNAJC4	0.045	-1.499
A_32_P97471	AK027225	AK027225	0.048	-1.499
A_24_P128977	NM_203505	G3BP2	0.012	-1.508
A_23_P153583	NM_006247	PPP5C	0.033	-1.510
A_24_P294199	NM_006226	PLCL1	0.019	-1.513
A_24_P409219	NM_017667	FLJ20097	0.038	-1.513
A_23_P200560	NM_001791	CDC42	0.011	-1.516
A_23_P319133	NM_018981	DNAJC10	0.045	-1.518
A_23_P39050	ENST00000341191	ENST00000341191	0.050	-1.521
A_23_P381449	NM_003110	SP2	0.040	-1.535
A_23_P123172	NR_002157	OR2A9P	0.028	-1.537
A_23_P208293	CR611629	CR611629	0.042	-1.543
A_23_P43765	NM_033540	MFN1	0.042	-1.547
A_32_P208321	THC2379531	THC2379531	0.041	-1.547
A_23_P251293	NM_003087	SNCG	0.032	-1.547
A_23_P134627	NM_133457	EMID2	0.045	-1.549
A_32_P540407	NM_207333	ZNF320	0.035	-1.552
A_24_P56833	NM_000413	HSD17B1	0.043	-1.552
A_32_P6535	XM_934128	PLGLA1	0.025	-1.567
A_24_P143189	NM_183049	TMSL3	0.043	-1.571
A_24_P28524	NM_001918	DBT	0.049	-1.574
A_23_P39263	NM_173480	ZNF57	0.006	-1.574
A_32_P200669	THC2408235	THC2408235	0.049	-1.578
A_23_P420092	AL833317	DENND4A	0.018	-1.578
A_32_P131940	NM_172020	POM121	0.049	-1.581
A_32_P121674	A_32_P121674	A_32_P121674	0.047	-1.581
A_24_P247749	NM_014999	RAB21	0.031	-1.582
A_24_P714707	A_24_P714707	A_24_P714707	0.048	-1.584
A_23_P305335	BC017770	BC017770	0.000	-1.589
A_23_P133263	NM_002130	HMGCS1	0.021	-1.592
A_23_P165390	NM_017964	SLC30A6	0.041	-1.597

A_32_P35441	BX104176	BX104176	0.019	-1.601
A_23_P55639	NM_018170	P15RS	0.035	-1.606
A_32_P148627	AX775927	AX775927	0.037	-1.616
A_24_P123155	NM_016331	ZNF639	0.041	-1.618
A_24_P13024	AK124901	SLC16A12	0.022	-1.621
A_23_P115645	NM_006561	CUGBP2	0.022	-1.623
A_32_P223644	NM_025083	EDC3	0.004	-1.625
A_23_P120316	NM_006636	MTHFD2	0.020	-1.636
A_32_P213822	AK025762	AK025762	0.034	-1.637
A_24_P291680	NM_144571	CNOT6L	0.014	-1.638
A_24_P145047	NM_015042	ZNF609	0.043	-1.638
A_32_P207767	THC2436597	THC2436597	0.011	-1.647
A_23_P141447	NM_145654	RDM1	0.012	-1.667
A_23_P62932	NM_001677	ATP1B1	0.004	-1.672
A_23_P10081	NM_182896	ARL13B	0.004	-1.674
A_23_P41470	NM_017631	FLJ20035	0.050	-1.684
A_23_P110151	NM_031305	ARHGAP24	0.030	-1.685
A_23_P105535	NM_172240	WDR51B	0.004	-1.690
A_23_P254852	NM_194248	OTOF	0.009	-1.696
A_23_P209337	NM_145280	FAM119A	0.033	-1.697
A_23_P74794	NM_002858	ABCD3	0.035	-1.699
A_23_P104651	NM_080668	CDCA5	0.044	-1.702
A_23_P98571	NM_145017	C11orf66	0.040	-1.704
A_23_P211773	NM_012287	CENTB2	0.015	-1.712
A_24_P387321	NM_016264	ZNF44	0.041	-1.740
A_24_P364838	NM_004785	SLC9A3R2	0.012	-1.742
A_23_P134204	NM_032599	NYD-SP18	0.033	-1.749
A_24_P710270	THC2440422	THC2440422	0.049	-1.753
A_24_P137897	NM_001007245	IFRD1	0.027	-1.757
A_23_P208090	NM_015846	MBD1	0.033	-1.770
A_32_P75867	THC2376729	THC2376729	0.029	-1.775
A_24_P205242	BC034378	RPL23	0.004	-1.776
A_23_P65618	NM_000359	TGM1	0.005	-1.777
A_23_P81660	NM_018368	LMBRD1	0.027	-1.779
A_23_P101351	NM_024106	ZNF426	0.046	-1.791
A_32_P51119	NM_152709	STOX1	0.023	-1.798
A_24_P388860	NM_003999	OSMR	0.043	-1.806
A_24_P153643	NM_004947	DOCK3	0.046	-1.813
A_32_P335921	NM_031916	ROPN1L	0.025	-1.814
A_23_P76435	NM_176818	15E1.2	0.046	-1.821
A_24_P237613	NM_182488	USP12	0.032	-1.829
A_23_P385861	NM_152562	CDCA2	0.013	-1.830
A_24_P250614	NM_005877	SF3A1	0.024	-1.832
A_23_P67278	NM_005815	ZNF443	0.024	-1.834
A_32_P722809	BC034142	IGKV1-5	0.048	-1.842
A_32_P130522	THC2311764	THC2311764	0.036	-1.854
A_23_P254140	NM_032131	ARMC2	0.016	-1.858
A_23_P141917	NM_003331	TYK2	0.043	-1.860
A_32_P31757	BX094773	BX094773	0.050	-1.865
A_23_P73096	AW993939	AW993939	0.045	-1.876

A_24_P112110	NM_001518	GTF2I	0.015	-1.889
A_23_P78265	NM_004138	KRT33A	0.043	-1.894
A_32_P201616	THC2279840	THC2279840	0.030	-1.898
A_24_P920025	NM_001039462	C8orf79	0.014	-1.906
A_32_P205895	BF089603	BF089603	0.039	-1.953
A_23_P302634	NM_173591	FLJ90579	0.006	-1.966
A_32_P115358	NM_023075	MPPE1	0.048	-1.979
A_23_P63655	NM_005174	ATP5C1	0.042	-1.982
A_23_P25945	NM_020421	ADCK1	0.046	-2.009
A_24_P73817	NM_001039503	LOC339105	0.030	-2.085
A_23_P55198	NM_173478	CNTD1	0.000	-2.118
A_24_P350196	CR600908	CR600908	0.014	-2.124
A_24_P333341	AK074586	SCAMP4	0.047	-2.130
A_32_P16918	NM_019596	C21orf62	0.040	-2.148
A_23_P171324	NM_024003	L1CAM	0.010	-2.156
A_24_P686965	AK124869	SH2D5	0.028	-2.158
A_24_P129205	NM_012160	FBXL4	0.049	-2.205
A_23_P113762	A_23_P113762	A_23_P113762	0.018	-2.260
A_24_P210406	NM_001730	KLF5	0.041	-2.734
A_23_P121596	NM_002704	PPBP	0.033	-2.856
A_23_P161209	NM_004747	DLG5	0.047	-3.598
A_24_P627503	ENST00000379900	ENST00000379900	0.017	-3.659

Group 2 Comparison				
ProbeName	SystematicName	GeneName	p value	Fold (t12 / t0)
A_23_P99275	NM_002258	KLRB1	0.035	8.404
A_23_P206806	NM_002209	ITGAL	0.035	6.403
A_32_P169550	AW977527	AW977527	0.036	5.424
A_23_P145718	NM_001637	AOAH	0.038	5.422
A_23_P91095	NM_006139	CD28	0.043	5.276
A_24_P683861	ENST00000361266	ENST00000361266	0.048	4.988
A_23_P144274	NM_144720	JAKMIP1	0.024	4.525
A_24_P12397	NM_018965	TREM2	0.034	4.421
A_24_P262688	NM_021706	LAIR1	0.033	4.290
A_23_P500433	NM_052813	CARD9	0.012	4.284
A_23_P128808	NM_013345	GPR132	0.042	4.263
A_32_P98162	AA019203	AA019203	0.026	3.956
A_23_P254507	NM_139211	HOP	0.009	3.874
A_32_P188263	AW383740	AW383740	0.005	3.749
A_23_P154367	NM_004226	STK17B	0.044	3.708
A_23_P201160	CR608653	CD1E	0.007	3.662
A_23_P86931	NM_198277	SLC37A2	0.007	3.551
A_23_P73837	NM_016610	TLR8	0.015	3.513
A_32_P49668	BC111482	BC111482	0.011	3.345
A_32_P132300	AA731356	AA731356	0.005	3.249
A_24_P938284	ENST00000367960	ENST00000367960	0.023	3.224
A_24_P224926	NM_002405	MFNG	0.039	3.124
A_24_P846810	A_24_P846810	A_24_P846810	0.045	3.067
A_23_P208182	NM_033130	SIGLEC10	0.007	3.044
A_32_P141338	BE930053	BE930053	0.049	2.930
A_23_P56356	NM_153021	PLB1	0.041	2.912
A_23_P101407	NM_000064	C3	0.002	2.897
A_32_P200025	A_32_P200025	A_32_P200025	0.049	2.878
A_32_P191417	AW276186	AW276186	0.028	2.866
A_23_P257542	NM_033054	MYO1G	0.018	2.858
A_24_P365975	NM_005202	COL8A2	0.007	2.845
A_24_P924957	L32537	KRT18	0.011	2.844
A_24_P42264	NM_000239	LYZ	0.026	2.843
A_32_P32315	A_32_P32315	A_32_P32315	0.001	2.841
A_24_P206343	NM_033054	MYO1G	0.009	2.792
A_24_P913930	BY798802	BY798802	0.010	2.692
A_23_P84084	NM_001495	GFRA2	0.003	2.687
A_23_P73348	NM_024335	IRX6	0.041	2.668
A_23_P56746	NM_004460	FAP	0.042	2.660
A_24_P52733	NM_001558	IL10RA	0.038	2.643
A_23_P6293	NM_018961	UBASH3A	0.049	2.622
A_23_P209006	NM_005543	INSL3	0.010	2.621
A_24_P54174	NM_001066	TNFRSF1B	0.042	2.574
A_32_P93309	CK824959	CK824959	0.034	2.545

A_24_P81900	NM_006931	SLC2A3	0.002	2.505
A_23_P149517	NM_002644	PIGR	0.030	2.497
A_23_P416747	NM_000733	CD3E	0.014	2.486
A_23_P321913	NM_022047	DEF6	0.013	2.480
A_23_P202327	NM_003474	ADAM12	0.007	2.469
A_24_P305345	NM_021155	CD209	0.017	2.438
A_32_P116488	THC2283842	THC2283842	0.037	2.438
A_32_P93668	BQ009471	BQ009471	0.033	2.433
A_24_P668974	CD048206	CD048206	0.042	2.424
A_23_P106362	NM_020980	AQP9	0.013	2.400
A_32_P99492	BX427588	BX427588	0.026	2.396
A_23_P61057	NM_004513	IL16	0.025	2.394
A_23_P70539	CR598918	HLA-C	0.046	2.374
A_23_P13382	NM_001013254	LSP1	0.042	2.357
A_23_P142447	NM_012335	MYO1F	0.016	2.349
A_23_P86283	NM_006762	LAPTM5	0.028	2.344
A_23_P26771	NM_006678	CD300C	0.005	2.336
A_23_P64044	NM_178443	URP2	0.006	2.336
A_24_P408704	NM_004946	DOCK2	0.033	2.334
A_23_P170574	NM_178310	SNAI3	0.003	2.306
A_24_P414187	NM_014869	IQSEC1	0.021	2.304
A_23_P129496	NM_001795	CDH5	0.049	2.304
A_23_P122937	NM_014800	ELMO1	0.037	2.282
A_23_P62652	NM_003037	SLAMF1	0.017	2.278
A_24_P367432	BC070333	IGHV1-69	0.026	2.276
A_23_P111888	NM_138455	CTHRC1	0.030	2.233
A_23_P350451	NM_001198	PRDM1	0.002	2.227
A_23_P140290	NM_021136	RTN1	0.036	2.217
A_23_P207520	Z74615	COL1A1	0.005	2.217
A_24_P114124	NM_006762	LAPTM5	0.037	2.216
A_23_P344000	NM_178537	B4GALNT4	0.005	2.213
A_32_P128496	T40959	T40959	0.006	2.199
A_23_P26824	NM_030576	LIMD2	0.035	2.194
A_23_P50571	NM_014219	KIR2DL2	0.042	2.193
A_23_P131208	NM_006186	NR4A2	0.027	2.178
A_24_P365767	NM_000397	CYBB	0.049	2.177
A_24_P65373	NM_000419	ITGA2B	0.025	2.134
A_23_P163787	NM_004530	MMP2	0.016	2.129
A_24_P376848	NM_031281	FCRL5	0.012	2.125
A_23_P114903	NM_002155	HSPA6	0.031	2.121
A_23_P200741	NM_001937	DPT	0.035	2.118
A_23_P148807	NM_003503	CDC7	0.021	2.116
A_24_P92624	A_24_P92624	A_24_P92624	0.045	2.113
A_23_P111672	NM_152829	TES	0.017	2.094
A_23_P109508	NM_000631	NCF4	0.002	2.081
A_23_P88831	NM_003983	SLC7A6	0.002	2.077

A_24_P136299	A_24_P136299	A_24_P136299	0.010	2.076
A_24_P27977	NM_001001188	TRPM2	0.047	2.064
A_24_P538557	AK026802	MERTK	0.003	2.062
A_24_P199774	NM_022164	TINAGL1	0.011	2.059
A_24_P113926	NM_001937	DPT	0.030	2.058
A_32_P453321	NM_001004307	MGC33556	0.034	2.051
A_32_P180395	AI937689	AI937689	0.042	2.046
A_23_P46356	NM_024575	TNFAIP8L2	0.033	2.042
A_23_P58251	NM_001014448	CPZ	0.013	2.041
A_24_P73599	NM_172217	IL16	0.050	2.041
A_23_P30900	BC008585	HLA-DQA1	0.020	2.031
A_23_P93898	NM_020369	FSCN3	0.019	2.027
A_32_P137745	AW298233	AW298233	0.018	2.010
A_23_P250413	NM_022141	PARVG	0.016	1.997
A_23_P257014	NM_005873	RGS19	0.000	1.996
A_23_P125977	NM_172369	C1QC	0.015	1.992
A_24_P918436	NM_152221	CSNK1E	0.027	1.989
A_23_P27556	NM_001974	EMR1	0.040	1.988
A_23_P101774	NM_000453	SLC5A5	0.049	1.988
A_23_P26583	AK027414	NOD27	0.032	1.984
A_23_P259561	THC2364841	THC2364841	0.046	1.983
A_23_P207037	NM_007261	CD300A	0.000	1.982
A_24_P852756	NM_020056	HLA-DQA2	0.008	1.970
A_24_P53215	NM_199242	UNC13D	0.017	1.968
A_24_P48856	NM_000071	CBS	0.042	1.964
A_23_P85693	NM_004120	GBP2	0.047	1.963
A_32_P148507	BE707436	BE707436	0.041	1.963
A_23_P114883	NM_002023	FMOD	0.003	1.961
A_24_P171268	NM_182665	RASSF5	0.038	1.960
A_24_P400573	NM_152468	TMC8	0.042	1.949
A_23_P138194	NM_000433	NCF2	0.028	1.948
A_24_P11825	NM_000647	CCR2	0.024	1.944
A_32_P6972	THC2341837	THC2341837	0.002	1.942
A_23_P17481	NM_023068	SIGLEC1	0.012	1.942
A_23_P502314	NM_078481	CD97	0.010	1.934
A_23_P358548	NM_199329	SLC43A3	0.019	1.933
A_23_P100660	NM_002615	SERPINF1	0.017	1.929
A_23_P19510	NM_182549	HLA-DQB2	0.036	1.922
A_23_P214499	NM_007048	BTN3A1	0.011	1.922
A_24_P107303	NM_001558	IL10RA	0.033	1.915
A_24_P928250	A_24_P928250	A_24_P928250	0.049	1.913
A_24_P134816	NM_182557	BCL9L	0.002	1.911
A_24_P85775	NM_004848	C1orf38	0.039	1.910
A_23_P64214	NM_004716	PCSK7	0.041	1.908
A_24_P595223	AK098753	C22orf35	0.046	1.908
A_24_P6381	NM_178276	SERINC5	0.020	1.901

A_23_P151820	NM_024832	RIN3	0.009	1.892
A_24_P550973	AK097411	FLJ40092	0.049	1.890
A_23_P156708	NM_019105	TNXB	0.037	1.890
A_24_P149036	NM_001387	DPYSL3	0.050	1.887
A_24_P222655	NM_015991	C1QA	0.048	1.883
A_23_P136026	AK128476	IGHA1	0.020	1.882
A_23_P385500	NM_178841	RNF166	0.049	1.876
A_23_P310956	NM_058175	COL6A2	0.010	1.866
A_23_P259141	NM_030776	ZBP1	0.003	1.863
A_24_P490109	A_24_P490109	A_24_P490109	0.048	1.862
A_24_P196827	NM_002122	HLA-DQA1	0.002	1.860
A_23_P44794	NM_138453	RAB3C	0.030	1.849
A_24_P32151	NM_182705	FAM101B	0.006	1.849
A_23_P67360	NM_020904	PLEKHA4	0.025	1.848
A_23_P14769	NM_002005	FES	0.041	1.838
A_23_P148121	ENST00000309295	ENST00000309295	0.037	1.836
A_23_P5281	NM_005583	LYL1	0.040	1.835
A_23_P142533	NM_000090	COL3A1	0.003	1.828
A_24_P406986	NM_199329	SLC43A3	0.032	1.827
A_23_P124837	NM_002332	LRP1	0.046	1.827
A_24_P248345	NM_015441	OLFML2B	0.016	1.827
A_23_P216176	NM_138715	MSR1	0.043	1.822
A_23_P161481	NM_014431	KIAA1274	0.038	1.820
A_23_P3792	NM_003486	SLC7A5	0.035	1.820
A_23_P139912	NM_002178	IGFBP6	0.042	1.812
A_24_P154080	NM_001397	ECE1	0.028	1.812
A_32_P86763	NM_004613	TGM2	0.006	1.810
A_23_P29495	NM_001904	CTNNB1	0.050	1.807
A_23_P86748	AF119906	AF119906	0.000	1.801
A_23_P42331	NM_145904	HMGA1	0.036	1.801
A_23_P218646	NM_032945	TNFRSF6B	0.028	1.795
A_24_P940288	CR749720	PGS1	0.024	1.795
A_32_P146871	A_32_P146871	A_32_P146871	0.003	1.790
A_23_P34744	NM_000396	CTSK	0.017	1.787
A_23_P207354	NM_016438	HIGD1B	0.010	1.779
A_23_P56328	NM_031310	PLVAP	0.031	1.779
A_23_P41765	NM_002198	IRF1	0.001	1.778
A_23_P421323	A_23_P421323	A_23_P421323	0.026	1.778
A_23_P162486	NM_002831	PTPN6	0.033	1.778
A_23_P251499	NM_002593	PCOLCE	0.040	1.777
A_23_P90497	NM_012276	LILRA4	0.014	1.773
A_24_P78531	NM_014358	CLEC4E	0.042	1.771
A_24_P68079	ENST00000301807	ENST00000301807	0.025	1.768
A_32_P128980	BC062780	BC062780	0.038	1.767
A_23_P29499	NM_001904	CTNNB1	0.018	1.759
A_23_P71928	NM_005489	SH2D3C	0.040	1.755

A_23_P209167	NM_005860	FSTL3	0.034	1.750
A_24_P237036	NM_003807	TNFSF14	0.004	1.750
A_23_P125423	NM_001733	C1R	0.031	1.747
A_23_P147665	NM_198474	OLFML1	0.038	1.741
A_23_P156890	NM_003206	TCF21	0.029	1.739
A_23_P105409	NM_006301	MAP3K12	0.032	1.739
A_23_P114649	NM_021933	RP5-1077B9.4	0.042	1.738
A_23_P417363	AK057267	RSNL2	0.001	1.737
A_23_P343719	NM_000932	PLCB3	0.032	1.731
A_23_P26439	NM_024043	DBNDD1	0.033	1.727
A_23_P214638	NM_006709	EHMT2	0.017	1.722
A_24_P232365	NM_019043	APBB1IP	0.035	1.721
A_24_P337592	NM_181531	BTN2A2	0.020	1.719
A_24_P307135	NM_019105	TNXB	0.043	1.710
A_24_P214858	NM_024807	TREML2	0.049	1.709
A_23_P300174	NM_173620	HEXDC	0.015	1.707
A_32_P208370	A_32_P208370	A_32_P208370	0.031	1.706
A_23_P77000	NM_014909	VASH1	0.018	1.699
A_23_P336708	NM_178502	DTX3	0.050	1.694
A_23_P101025	NM_001040078	LOC654346	0.040	1.692
A_23_P384965	THC2314039	THC2314039	0.033	1.689
A_24_P126731	A_24_P126731	A_24_P126731	0.008	1.686
A_23_P210726	NM_021873	CDC25B	0.029	1.685
A_24_P803809	A_24_P803809	A_24_P803809	0.019	1.685
A_24_P794447	AK021552	LOC399959	0.005	1.683
A_24_P389409	NM_006129	BMP1	0.043	1.681
A_32_P187050	A_32_P187050	A_32_P187050	0.032	1.678
A_24_P123632	AF034174	AF034174	0.017	1.673
A_24_P12149	NM_014666	CLINT1	0.029	1.671
A_24_P101771	A_24_P101771	A_24_P101771	0.001	1.666
A_24_P297119	NM_138983	OLIG1	0.022	1.664
A_24_P22488	NM_003502	AXIN1	0.040	1.664
A_24_P757255	ENST00000377411	ENST00000377411	0.042	1.663
A_23_P2960	NM_005163	AKT1	0.027	1.654
A_23_P156788	NM_003764	STX11	0.043	1.652
A_24_P102283	AK098835	AK098835	0.004	1.652
A_23_P104881	NM_019055	ROBO4	0.023	1.649
A_23_P250866	NM_030935	TSC22D4	0.047	1.644
A_23_P52939	NM_003627	SLC43A1	0.022	1.634
A_24_P20292	CR626252	CR626252	0.016	1.632
A_23_P24077	NM_022153	C10orf54	0.033	1.632
A_24_P48187	NM_020753	CASKIN2	0.022	1.631
A_32_P218228	NM_001002034	FAM109B	0.006	1.626
A_23_P58869	NR_002932	LOC442245	0.027	1.621
A_23_P387057	NM_178014	TUBB	0.016	1.620
A_24_P317622	NM_031946	CENTG3	0.039	1.617

A_24_P263767	ENST00000376793	ENST00000376793	0.007	1.616
A_24_P935491	NM_000090	COL3A1	0.015	1.612
A_23_P353726	NM_153810	C10orf46	0.032	1.611
A_23_P774	ENST00000368646	ENST00000368646	0.019	1.610
A_23_P62583	NM_001409	MEGF6	0.039	1.605
A_24_P117053	NM_024509	LRFN3	0.004	1.603
A_32_P149011	CN479126	CN479126	0.035	1.602
A_23_P79134	NM_024310	PLEKHF1	0.026	1.602
A_23_P56127	NM_019108	FLJ12886	0.029	1.600
A_32_P140139	NM_000129	F13A1	0.006	1.597
A_24_P461497	A_24_P461497	A_24_P461497	0.011	1.592
A_24_P33595	THC2307600	THC2307600	0.026	1.586
A_23_P131614	NM_004369	COL6A3	0.011	1.580
A_23_P354953	NM_013433	TNPO2	0.042	1.572
A_23_P323836	NM_173691	C9orf75	0.032	1.572
A_23_P306148	NM_002675	PML	0.013	1.571
A_24_P349869	A_24_P349869	A_24_P349869	0.016	1.571
A_24_P615207	THC2340864	THC2340864	0.034	1.570
A_24_P252020	NM_033549	TRIM41	0.031	1.569
A_23_P80008	NM_033118	MYLK2	0.040	1.568
A_24_P376483	NM_002116	HLA-A	0.011	1.567
A_24_P305662	NM_022727	HTF9C	0.024	1.566
A_23_P57784	NM_021101	CLDN1	0.018	1.566
A_23_P47709	NM_000803	FOLR2	0.033	1.559
A_23_P430381	NM_212551	LYSMD1	0.003	1.557
A_23_P141055	NM_015927	TGFB1I1	0.029	1.555
A_23_P12549	NM_019043	APBB1IP	0.028	1.554
A_23_P73787	NM_153183	NUDT10	0.014	1.548
A_24_P77432	NM_133631	ROBO1	0.038	1.547
A_23_P397055	NM_019015	CSGlcA-T	0.022	1.541
A_24_P386334	NM_020145	SH3GLB2	0.006	1.540
A_24_P238744	BC092424	BC092424	0.005	1.540
A_23_P5938	AB020696	C20orf117	0.036	1.539
A_23_P202881	NM_005103	FEZ1	0.037	1.539
A_23_P212126	NM_080542	COLQ	0.039	1.539
A_32_P85591	NM_194324	MGC39900	0.031	1.537
A_23_P126398	NM_012432	SETDB1	0.038	1.537
A_23_P369960	NM_014551	hCAP-H2	0.040	1.536
A_23_P133506	NM_024872	DOK3	0.009	1.534
A_32_P139302	AI287491	AI287491	0.038	1.534
A_23_P24555	NM_015157	PHLDB1	0.029	1.533
A_24_P941912	NM_138287	DTX3L	0.033	1.531
A_24_P161933	CR608347	CR608347	0.015	1.531
A_23_P25587	NM_007015	LECT1	0.038	1.529
A_24_P298894	A_24_P298894	A_24_P298894	0.032	1.528
A_24_P735306	THC2338185	THC2338185	0.011	1.528

A_24_P56363	ENST00000355854	ENST00000355854	0.042	1.528
A_24_P605612	NM_003247	THBS2	0.003	1.526
A_23_P162386	NM_016293	BIN2	0.032	1.524
A_24_P398432	NM_139181	CENTD2	0.024	1.523
A_23_P207003	NM_004574	4-Sep	0.005	1.523
A_32_P26103	NM_006412	AGPAT2	0.008	1.522
A_24_P70993	NM_002414	CD99	0.016	1.521
A_23_P62959	NM_012396	PHLDA3	0.039	1.518
A_24_P43681	NM_014063	DBNL	0.026	1.516
A_23_P41066	NM_170717	RASSF1	0.045	1.516
A_24_P223518	NM_007270	FKBP9	0.022	1.516
A_24_P45651	NM_001005464	HIST2H3A	0.025	1.513
A_24_P387158	NM_005782	THOC4	0.001	1.512
A_24_P915227	AY129015	AY129015	0.027	1.510
A_23_P48691	NM_001663	ARF6	0.011	1.510
A_23_P137689	NM_015441	OLFML2B	0.038	1.509
A_23_P33433	NM_002383	MAZ	0.026	1.508
A_23_P150394	NM_022003	FXYD6	0.042	1.506
A_32_P146286	ENST00000339892	ENST00000339892	0.017	1.504
A_24_P571824	A_24_P571824	A_24_P571824	0.042	1.503
A_32_P147241	NM_182470	PKM2	0.037	1.502
A_24_P410017	ENST00000375923	ENST00000375923	0.014	1.501
A_23_P3312	NM_005545	ISLR	0.023	1.500
A_23_P150281	AK096176	TP53I11	0.014	1.499
A_24_P596251	A_24_P596251	A_24_P596251	0.005	1.498
A_24_P291278	AK000347	LAX1	0.028	1.497
A_23_P14853	NM_002344	LTK	0.022	1.495
A_24_P203315	NM_182553	CNIH2	0.012	1.494
A_23_P208389	NM_021913	AXL	0.039	1.493
A_24_P172481	NM_006074	TRIM22	0.018	1.492
A_23_P142289	NM_002067	GNA11	0.041	1.492
A_24_P65616	NM_006505	PVR	0.043	1.491
A_32_P107002	NM_001012391	LOC400509	0.006	1.491
A_23_P67339	NM_020650	RCN3	0.021	1.490
A_24_P247879	AK097244	PIGG	0.041	1.489
A_24_P65597	CR616003	CR616003	0.029	1.489
A_24_P110668	NM_020239	CDC42SE1	0.027	1.489
A_24_P237753	NM_015354	NUP188	0.014	1.488
A_24_P321093	NM_014767	SPOCK2	0.048	1.487
A_23_P201778	NM_080588	PTPN7	0.006	1.483
A_23_P337033	NM_012384	GMEB2	0.003	1.481
A_24_P255786	A_24_P255786	A_24_P255786	0.030	1.481
A_23_P335813	NM_016272	TOB2	0.019	1.481
A_24_P121535	NM_005159	ACTC	0.036	1.479
A_24_P306614	A_24_P306614	A_24_P306614	0.009	1.479
A_23_P340848	NM_000960	PTGIR	0.014	1.477

A_23_P27688	BC022233	BC022233	0.018	1.477
A_23_P152984	NM_005782	THOC4	0.037	1.476
A_24_P289376	NM_001013649	LOC388969	0.020	1.472
A_24_P94419	NM_015147	CEP68	0.008	1.471
A_24_P6903	NM_001017992	DKFZp686D0972	0.003	1.471
A_23_P342165	AK093019	FLJ35700	0.012	1.468
A_23_P149992	NM_020992	PDLIM1	0.048	1.467
A_23_P149019	NM_001703	BAI2	0.037	1.465
A_23_P201295	NM_017766	CASZ1	0.041	1.465
A_23_P119266	NM_001375	DNASE2	0.018	1.462
A_24_P414045	NM_006302	GCS1	0.035	1.462
A_23_P119448	NM_014931	SAPS1	0.015	1.462
A_24_P161393	A_24_P161393	A_24_P161393	0.036	1.461
A_24_P246107	NM_181600	KRTAP13-4	0.000	1.451
A_24_P412734	NM_173502	PRSS36	0.002	1.451
A_24_P187626	A_24_P187626	A_24_P187626	0.015	1.447
A_24_P941845	AK024319	GMPPB	0.005	1.445
A_23_P200126	NM_003684	MKNK1	0.022	1.444
A_23_P122579	NM_001350	DAXX	0.032	1.444
A_32_P113533	ENST00000312847	ENST00000312847	0.011	1.443
A_23_P139547	NM_006009	TUBA3	0.023	1.442
A_23_P100795	NM_213662	STAT3	0.029	1.440
A_32_P233379	THC2379201	THC2379201	0.048	1.440
A_23_P501276	NM_001069	TUBB2A	0.017	1.439
A_23_P88880	NM_015069	ZNF423	0.039	1.437
A_24_P49800	A_24_P49800	A_24_P49800	0.046	1.434
A_23_P202708	NM_003682	MADD	0.040	1.433
A_23_P31686	NM_021174	KIAA1967	0.016	1.428
A_32_P155776	NM_001017421	FKSG30	0.018	1.422
A_23_P381461	NM_144999	LRRC45	0.000	1.421
A_23_P71864	NM_032809	FAM73B	0.029	1.420
A_23_P347432	NM_181870	DVL1	0.004	1.418
A_23_P45913	NM_004814	WDR57	0.033	1.417
A_23_P421935	BC037418	ATXN7L3	0.011	1.416
A_23_P206684	NM_199424	WWP2	0.046	1.416
A_23_P203420	NM_004739	MTA2	0.021	1.414
A_23_P128161	NM_032704	TUBA6	0.002	1.414
A_23_P58482	NM_002406	MGAT1	0.005	1.414
A_23_P252721	NM_182643	DLC1	0.036	1.412
A_23_P149075	NM_018067	RPRC1	0.023	1.409
A_24_P889070	AK023647	AK023647	0.046	1.407
A_24_P911112	AK124344	AK124344	0.041	1.404
A_24_P850172	XM_926717	LOC643386	0.010	1.403
A_23_P11936	NM_145345	UBXD5	0.036	1.400
A_24_P578445	A_24_P578445	A_24_P578445	0.041	1.399
A_32_P22401	NM_018067	RPRC1	0.042	1.398

A_23_P49546	NM_000835	GRIN2C	0.013	1.396
A_24_P23034	NM_021035	ZNFX1	0.025	1.392
A_23_P139123	NM_000062	SERPING1	0.015	1.387
A_24_P201089	NM_016612	SLC25A37	0.014	1.386
A_23_P38468	NM_014604	TAX1BP3	0.048	1.386
A_23_P150718	NM_001665	RHOG	0.023	1.386
A_23_P159920	NM_003639	IKBKG	0.022	1.386
A_24_P210829	NM_005009	NME4	0.004	1.384
A_23_P407684	NM_178167	ZNF598	0.045	1.383
A_23_P150249	NM_006848	CCDC85B	0.031	1.382
A_24_P584936	BE243347	BE243347	0.043	1.381
A_23_P118406	NM_001033046	C17orf62	0.036	1.380
A_24_P226554	NM_001101	ACTB	0.017	1.379
A_24_P749042	A_24_P749042	A_24_P749042	0.005	1.379
A_23_P343303	NM_004188	GFI1B	0.011	1.378
A_23_P210425	NM_181526	MYL9	0.010	1.377
A_24_P16071	A_24_P16071	A_24_P16071	0.017	1.377
A_23_P416468	NM_025049	C15orf20	0.043	1.373
A_32_P14544	NM_002952	RPS2	0.044	1.371
A_24_P242138	NM_182614	FAM70B	0.016	1.370
A_23_P202156	NM_002502	NFKB2	0.006	1.369
A_24_P561165	A_24_P561165	A_24_P561165	0.030	1.364
A_32_P420563	NM_001017981	LOC200312	0.034	1.363
A_23_P312344	NM_016196	RBM19	0.001	1.363
A_23_P381979	NM_181672	OGT	0.028	1.360
A_23_P46131	NM_024869	GRRP1	0.002	1.360
A_23_P213394	NM_182925	FLT4	0.022	1.358
A_23_P3483	NM_021098	CACNA1H	0.010	1.356
A_32_P409222	NM_033113	ZNF628	0.011	1.355
A_24_P7021	A_24_P7021	A_24_P7021	0.032	1.352
A_23_P351837	NM_001039548	FLJ33790	0.042	1.351
A_23_P141429	NM_016428	ABI3	0.027	1.350
A_24_P374652	NM_022731	NUCKS1	0.021	1.349
A_23_P355471	NM_001060	TBXA2R	0.047	1.348
A_24_P211558	NM_032051	ZNF278	0.044	1.345
A_24_P322395	NM_080732	EGLN2	0.005	1.344
A_23_P144151	AF292100	DCUN1D1	0.030	1.343
A_23_P352870	NM_002856	PVRL2	0.024	1.342
A_24_P339272	NM_152350	C17orf45	0.034	1.341
A_23_P130648	NM_005716	GIPC1	0.031	1.341
A_24_P829183	AK056817	FLJ32255	0.007	1.339
A_24_P396650	NM_002953	RPS6KA1	0.013	1.338
A_23_P251898	NM_152743	C7orf27	0.005	1.337
A_24_P255628	NM_015197	PACS2	0.045	1.337
A_24_P57426	NM_030582	COL18A1	0.030	1.337
A_32_P15320	NM_001402	EEF1A1	0.004	1.335

A_24_P7594	NM_030641	APOL6	0.025	1.335
A_32_P412313	NM_017873	ASB6	0.040	1.330
A_32_P120014	A_32_P120014	A_32_P120014	0.050	1.329
A_23_P39131	NM_015710	GLTSCR2	0.032	1.328
A_23_P37988	NM_152727	CPNE2	0.022	1.327
A_23_P51136	NM_004040	RHOB	0.038	1.325
A_32_P34186	NM_001417	EIF4B	0.024	1.323
A_24_P159515	NM_014726	TBKBP1	0.032	1.321
A_23_P75989	NM_002576	PAK1	0.032	1.320
A_32_P126928	AF119875	IER3IP1	0.021	1.319
A_23_P61230	NM_004712	HGS	0.030	1.319
A_23_P48109	NM_016533	NINJ2	0.023	1.318
A_23_P108376	NM_022065	THADA	0.017	1.315
A_23_P146981	A_23_P146981	A_23_P146981	0.013	1.314
A_23_P124559	NM_031300	MXD3	0.036	1.312
A_23_P153489	NM_002975	CLEC11A	0.017	1.311
A_24_P924202	BX640978	PCNXL3	0.028	1.307
A_23_P91615	ENST00000333131	ENST00000333131	0.001	1.305
A_23_P132260	NM_014303	PES1	0.043	1.305
A_32_P184727	NM_002265	KPNB1	0.040	1.305
A_24_P247175	A_24_P247175	A_24_P247175	0.024	1.303
A_23_P152963	NM_017622	C17orf59	0.020	1.303
A_24_P58529	NM_032704	TUBA6	0.005	1.303
A_24_P416059	NM_006715	MAN2C1	0.033	1.301
A_32_P188388	AA586832	AA586832	0.049	1.297
A_24_P323795	A_24_P323795	A_24_P323795	0.026	1.296
A_23_P210395	NM_000744	CHRNA4	0.040	1.296
A_23_P26476	NM_032259	WDR24	0.041	1.292
A_24_P323901	ENST00000338302	ENST00000338302	0.003	1.292
A_23_P307430	NM_152344	LSM12	0.027	1.290
A_23_P1387	NM_032900	ARHGAP19	0.043	1.289
A_23_P156928	NM_001503	GPLD1	0.047	1.289
A_32_P24382	BC063625	KRTAP2-4	0.022	1.287
A_23_P404685	NM_178348	LCE1A	0.040	1.286
A_23_P74887	AB007937	SDC3	0.008	1.283
A_23_P152406	NM_032330	CAPNS2	0.007	1.282
A_23_P115190	NM_002506	NGFB	0.044	1.280
A_32_P20697	NM_003992	CLK3	0.029	1.280
A_23_P29079	NM_001002021	PFKL	0.022	1.279
A_32_P116113	CN479762	CN479762	0.047	1.279
A_24_P790909	AI652176	AI652176	0.026	1.277
A_23_P109072	NM_020436	SALL4	0.049	1.272
A_23_P81811	NM_080604	TJAP1	0.011	1.272
A_32_P30600	THC2284350	THC2284350	0.037	1.271
A_24_P93111	A_24_P93111	A_24_P93111	0.035	1.270
A_23_P337658	NM_001631	ALPI	0.019	1.269

A_23_P50368	NM_206818	OSCAR	0.013	1.269
A_24_P329939	NM_021729	VPS11	0.018	1.269
A_23_P67391	NM_007059	KPTN	0.010	1.268
A_23_P16683	NM_017722	TRMT1	0.027	1.268
A_23_P156732	NM_024165	PHF1	0.003	1.266
A_24_P73408	NM_145293	LOC196549	0.042	1.266
A_32_P233713	A_32_P233713	A_32_P233713	0.049	1.265
A_24_P106145	NM_017491	WDR1	0.043	1.262
A_24_P272290	NM_183373	C6orf145	0.041	1.262
A_24_P171274	NM_000427	LOR	0.007	1.261
A_23_P15414	NM_145351	SCARF1	0.023	1.261
A_23_P401580	NM_022762	RMND5B	0.046	1.260
A_23_P121265	NM_007284	PTK9L	0.009	1.259
A_23_P203505	NM_033034	TRIM5	0.019	1.257
A_23_P203419	NM_013402	FADS1	0.014	1.252
A_23_P112103	NM_024736	GSDMDC1	0.023	1.245
A_23_P400147	ENST00000263284	ENST00000263284	0.009	1.241
A_24_P833191	THC2366678	THC2366678	0.011	1.236
A_23_P74097	NM_003198	TCEB3	0.050	1.235
A_24_P285378	BC001256	C1orf91	0.028	1.234
A_24_P109432	ENST00000339968	ENST00000339968	0.033	1.233
A_23_P19004	NM_023924	BRD9	0.033	1.228
A_24_P941167	NM_030641	APOL6	0.038	1.228
A_23_P9768	NM_053051	CNTROB	0.006	1.228
A_23_P30848	NM_005516	HLA-E	0.006	1.227
A_23_P142304	NM_017572	MKNK2	0.019	1.226
A_23_P84565	NM_001722	POLR3D	0.003	1.226
A_23_P142389	NM_205834	LSR	0.009	1.224
A_23_P74653	NM_006600	NUDC	0.027	1.220
A_32_P23517	BU753102	BU753102	0.042	1.218
A_23_P88971	NM_000086	CLN3	0.013	1.217
A_24_P230734	AK091468	SMUG1	0.045	1.215
A_32_P44620	AK123438	AK123438	0.034	1.214
A_32_P88719	NM_080879	RAB40A	0.047	1.212
A_23_P41872	NM_007255	B4GALT7	0.028	1.210
A_23_P164883	NM_007254	PNKP	0.038	1.206
A_23_P41987	NM_001496	GFRA3	0.029	1.204
A_23_P43369	NM_014450	SIT1	0.039	1.203
A_24_P207139	NM_033238	PML	0.042	1.200
A_24_P187197	BX648930	BX648930	0.032	1.195
A_32_P204218	NM_205855	UNQ1940	0.019	1.194
A_32_P148118	ENST00000331696	ENST00000331696	0.016	1.192
A_24_P117368	AK055306	AK055306	0.035	1.192
A_24_P714750	A_24_P714750	A_24_P714750	0.024	1.190
A_23_P9036	NM_015117	ZC3H3	0.033	1.189
A_23_P40347	NM_178580	HM13	0.015	1.185

A_23_P95165	NM_020210	SEMA4B	0.019	1.185
A_24_P136453	NM_022648	TNS1	0.046	1.173
A_23_P36345	NM_006645	STARD10	0.010	1.167
A_23_P122116	NM_016222	DDX41	0.016	1.166
A_24_P308029	NM_144617	HSPB6	0.049	1.164
A_24_P155815	NM_032382	COG8	0.027	1.161
A_24_P185624	NM_173179	SLC35C2	0.041	1.160
A_23_P26640	NM_152459	MGC45438	0.043	1.159
A_24_P91238	NM_032837	FAM104A	0.001	1.157
A_23_P326844	NM_174913	C14orf21	0.019	1.153
A_24_P404593	NM_177405	CECR1	0.030	1.148
A_24_P913210	AF064484	SLC11A2	0.003	1.148
A_23_P145006	NM_054023	SCGB3A2	0.044	1.140
A_24_P341517	ENST00000317656	ENST00000317656	0.005	1.137
A_24_P928658	NM_005411	SFTPA1	0.045	1.136
A_24_P280660	NM_178174	TREML1	0.010	1.132
A_32_P234459	NR_001434	HLA-H	0.013	1.131
A_32_P230736	XM_374010	LOC389033	0.016	1.127
A_23_P70814	NM_014356	C6orf123	0.037	1.126
A_23_P128337	NM_002824	PTMS	0.027	1.121
A_32_P223488	THC2372788	THC2372788	0.040	1.120
A_23_P164999	NM_004359	CDC34	0.012	1.112
A_24_P52168	A_24_P52168	A_24_P52168	0.005	1.111
A_24_P917951	BC009786	NOC2L	0.039	1.110
A_24_P82848	NM_138422	LOC113179	0.003	1.102
A_23_P350782	ENST00000307840	ENST00000307840	0.032	1.098
A_32_P116203	NM_000265	NCF1	0.049	1.096
A_23_P254442	NM_001004302	LOC155060	0.039	1.091
A_23_P168551	NM_153247	SLC29A4	0.011	1.078
A_23_P161280	NM_014767	SPOCK2	0.044	1.072
A_24_P49183	NM_017820	FLJ20433	0.009	1.064
A_23_P155807	AY629351	ZCCHC4	0.015	1.063
A_23_P97541	NM_000715	C4BPA	0.016	1.062
A_23_P153390	NM_198492	CLEC4G	0.025	1.018
A_23_P1759	NM_153206	AMICA1	0.040	-1.007
A_23_P368101	NM_024632	SAP30L	0.041	-1.020
A_24_P219785	NM_005184	CALM3	0.042	-1.035
A_23_P109452	NM_001005735	CHEK2	0.014	-1.058
A_24_P154006	NM_005005	NDUFB9	0.028	-1.058
A_23_P1638	NM_031492	RBM4B	0.000	-1.074
A_24_P246841	NM_004277	SLC25A27	0.045	-1.077
A_24_P301655	NM_001772	CD33	0.047	-1.099
A_24_P509177	BC073157	LOC92482	0.042	-1.101
A_23_P97932	NM_012228	MSRB2	0.041	-1.118
A_23_P202374	NM_078470	COX15	0.031	-1.133
A_24_P565898	A_24_P565898	A_24_P565898	0.045	-1.135

A_24_P217848	NM_003510	HIST1H2AK	0.007	-1.135
A_23_P422115	NM_144654	C9orf116	0.038	-1.136
A_23_P317207	NM_153340	ATXN7L2	0.041	-1.160
A_23_P165086	NM_024407	NDUFS7	0.042	-1.161
A_32_P113007	THC2415754	THC2415754	0.025	-1.164
A_24_P418536	A_24_P418536	A_24_P418536	0.020	-1.169
A_23_P11017	AK021866	AK021866	0.021	-1.174
A_23_P363196	NM_014418	TCL6	0.006	-1.183
A_32_P7204	NM_004249	RAB28	0.043	-1.195
A_23_P21713	NM_024532	SPAG16	0.037	-1.210
A_23_P41824	A_23_P41824	A_23_P41824	0.037	-1.225
A_23_P213718	NM_014402	UQCRQ	0.035	-1.230
A_23_P160668	NM_005376	MYCL1	0.030	-1.232
A_24_P272073	ENST00000335078	ENST00000335078	0.039	-1.234
A_23_P78061	NM_005568	LHX1	0.042	-1.237
A_32_P49334	BC036909	LOC284889	0.001	-1.262
A_23_P15542	NM_000413	HSD17B1	0.031	-1.274
A_24_P243329	NM_002203	ITGA2	0.039	-1.303
A_24_P281923	NM_001931	DLAT	0.046	-1.315
A_32_P224855	NM_024646	ZYG11B	0.035	-1.359
A_23_P355439	NM_170745	HIST1H2AA	0.011	-1.360
A_24_P915355	NM_181489	ZNF445	0.027	-1.362
A_24_P602348	THC2439499	THC2439499	0.048	-1.369
A_24_P410286	AJ291670	DMRTC1	0.047	-1.373
A_23_P201461	NM_016831	PER3	0.040	-1.375
A_32_P201616	THC2279840	THC2279840	0.030	-1.386
A_23_P111194	NM_012391	SPDEF	0.006	-1.400
A_32_P193218	AK123506	AK123506	0.031	-1.409
A_32_P135489	A_32_P135489	A_32_P135489	0.033	-1.412
A_23_P351535	AF229804	LOC642378	0.049	-1.421
A_32_P201041	AK124956	AK124956	0.041	-1.438
A_24_P922969	NM_194301	GARNL1	0.018	-1.445
A_23_P417237	NM_020177	FEM1C	0.025	-1.445
A_23_P94133	NM_015029	POP1	0.043	-1.446
A_23_P340698	NM_002426	MMP12	0.038	-1.462
A_24_P379727	NM_032309	CHCHD5	0.038	-1.473
A_24_P145377	NM_005348	HSP90AA1	0.048	-1.485
A_24_P230708	NM_177966	2'-PDE	0.042	-1.512
A_32_P158786	ENST00000333722	ENST00000333722	0.001	-1.523
A_23_P368624	NM_000748	CHRNB2	0.037	-1.559
A_24_P135276	NM_032172	USP42	0.010	-1.563
A_23_P420942	AF495759	MT1E	0.025	-1.610
A_23_P102607	ENST00000373219	ENST00000373219	0.049	-1.617
A_23_P120883	NM_002133	HMOX1	0.048	-1.621
A_23_P407750	A_23_P407750	A_23_P407750	0.027	-1.642
A_32_P216520	NM_007191	WIF1	0.009	-1.679

A_24_P361896	NM_005953	MT2A	0.039	-1.680
A_23_P252413	ENST00000245185	ENST00000245185	0.048	-1.715
A_24_P307674	AK024051	LRRC41	0.039	-1.744
A_23_P66241	NM_176870	MT1M	0.014	-1.790
A_32_P187349	THC2415323	THC2415323	0.026	-1.833
A_23_P65254	NM_015932	POMP	0.027	-1.892
A_23_P52639	NM_004074	COX8A	0.032	-2.027
A_24_P631625	A_24_P631625	A_24_P631625	0.039	-2.110
A_23_P384532	NM_145020	CCDC11	0.047	-2.243
A_24_P721815	AK002066	AK002066	0.031	-2.295
A_23_P108280	NM_023944	CYP4F12	0.034	-2.395
A_24_P660811	NM_001013651	LOC389607	0.048	-2.427
A_23_P15174	NM_005949	MT1F	0.005	-2.512
A_32_P37143	BX649059	GAS2L3	0.037	-3.801

t0 Comparison				
**ProbeName**	**SystematicName**	**GeneName**	**p value**	**Fold (Very High / High)**
A_24_P264192	NM_005839	SRRM1	0.0004	23.92
A_24_P281801	ENST00000341087	ENST00000341087	0.0062	18.31
A_24_P3804	NM_001039651	C6orf26	0.0459	17.17
A_24_P211351	ENST00000370395	ENST00000370395	0.0290	16.33
A_23_P116913	NM_153207	AEBP2	0.0204	16.32
A_23_P501996	NM_199203	Kua-UEV	0.0135	15.47
A_23_P71644	NM_004629	FANCG	0.0165	14.98
A_24_P187787	NM_014159	SETD2	0.0080	14.92
A_23_P71379	NM_005672	PSCA	0.0497	14.82
A_23_P43726	NM_015231	NUP160	0.0296	12.79
A_24_P68203	BC015216	LOC653242	0.0148	12.65
A_23_P45099	NM_002125	HLA-DRB5	0.0155	12.50
A_23_P204417	NM_018463	ITFG2	0.0263	12.17
A_23_P166663	NM_012096	APPL	0.0236	11.65
A_23_P82449	NM_004403	DFNA5	0.0337	11.54
A_24_P753760	A_24_P753760	A_24_P753760	0.0123	11.32
A_24_P350589	NM_020724	RNF150	0.0109	11.28
A_24_P199655	NM_138959	VANGL1	0.0095	10.85
A_32_P313405	NM_005559	LAMA1	0.0470	10.44
A_24_P158946	NM_139241	FGD4	0.0047	10.21
A_23_P139654	NM_007328	KLRC1	0.0137	10.12
A_24_P261734	CR594705	CR594705	0.0275	10.05
A_24_P306810	ENST00000319902	ENST00000319902	0.0054	10.03
A_23_P210719	NM_016143	NSFL1C	0.0259	9.12
A_24_P10137	NM_014059	RGC32	0.0220	9.05
A_24_P579984	AK057167	AK057167	0.0094	8.88
A_23_P330461	NM_144686	TMC4	0.0174	8.87
A_24_P24890	A_24_P24890	A_24_P24890	0.0073	8.63
A_24_P868583	NM_001012421	ANKRD20A2	0.0263	8.55
A_32_P2730	BC005081	BCAN	0.0152	8.40
A_24_P573514	BC033528	BC033528	0.0152	8.24
A_23_P6963	NM_003341	UBE2E1	0.0235	7.88
A_24_P145316	NM_183040	DTNBP1	0.0318	7.84
A_23_P216361	NM_021110	COL14A1	0.0111	7.81
A_24_P922684	BC020539	BC020539	0.0298	7.69
A_23_P502320	NM_001138	AGRP	0.0360	7.55
A_24_P204290	ENST00000342830	ENST00000342830	0.0154	7.46
A_23_P129786	NM_001005291	SREBF1	0.0429	7.45
A_32_P460399	AL365520	AL365520	0.0337	7.38
A_32_P92563	AK097893	LOC257396	0.0421	7.27
A_24_P401739	NM_001006634	ARHGAP17	0.0308	7.27
A_23_P47704	NM_003355	UCP2	0.0219	7.20
A_24_P315581	A_24_P315581	A_24_P315581	0.0428	6.97
A_23_P125016	A_23_P125016	A_23_P125016	0.0302	6.96

A_23_P217028	NM_001008563	USP20	0.0231	6.93
A_23_P23048	NM_002965	S100A9	0.0374	6.87
A_23_P75402	NM_004551	NDUFS3	0.0133	6.80
A_32_P138348	NM_017527	LY6K	0.0247	6.79
A_23_P17998	NM_005524	HES1	0.0258	6.76
A_23_P217832	NM_052966	C1orf24	0.0241	6.73
A_24_P401601	A_24_P401601	A_24_P401601	0.0221	6.73
A_23_P86917	NM_003824	FADD	0.0243	6.72
A_23_P207319	NM_003954	MAP3K14	0.0386	6.65
A_23_P60354	NM_139045	SMARCA2	0.0249	6.62
A_23_P339633	NM_174931	CCDC75	0.0244	6.59
A_24_P910580	NM_181077	GOLGA8A	0.0084	6.59
A_24_P394865	NM_016548	GOLPH2	0.0223	6.49
A_23_P217785	NM_016500	CXorf26	0.0361	6.49
A_24_P416961	NM_001670	ARVCF	0.0164	6.46
A_32_P140742	NM_001040710	FLJ30851	0.0283	6.37
A_23_P204640	NM_024865	NANOG	0.0446	6.31
A_23_P88331	NM_014750	DLG7	0.0493	6.30
A_23_P10614	NM_002610	PDK1	0.0482	6.28
A_23_P126426	NM_001384	DPH2	0.0287	6.23
A_24_P290527	NM_003410	ZFX	0.0355	6.23
A_23_P3562	NM_032178	SLC7A6OS	0.0484	6.21
A_23_P73311	NM_052958	C8orf34	0.0259	6.12
A_23_P363399	NM_030674	SLC38A1	0.0335	6.05
A_32_P54628	THC2446669	THC2446669	0.0324	5.97
A_32_P34750	AV702101	AV702101	0.0468	5.94
A_24_P937965	BC030992	BC030992	0.0041	5.89
A_24_P217063	THC2337363	THC2337363	0.0274	5.87
A_24_P163590	NM_152556	FLJ31818	0.0372	5.79
A_24_P247920	AB051439	KIAA1652	0.0063	5.63
A_24_P152743	AK021738	TMC6	0.0178	5.62
A_23_P413456	AK128423	CIRBP	0.0339	5.61
A_24_P727868	BE671816	BE671816	0.0393	5.48
A_23_P210131	ENST00000289105	ENST00000289105	0.0243	5.41
A_23_P351148	NM_053282	SH2D1B	0.0377	5.38
A_23_P319783	NM_206996	SPAG17	0.0037	5.34
A_23_P24751	NM_173810	TTC9C	0.0432	5.30
A_24_P326708	NM_003957	BRSK2	0.0452	5.30
A_23_P17269	NM_018084	KIAA1212	0.0457	5.28
A_24_P153576	NM_173082	SHPRH	0.0340	5.17
A_32_P122595	A_32_P122595	A_32_P122595	0.0233	5.15
A_24_P829261	NR_002819	MALAT1	0.0213	5.09
A_23_P4014	AY168921	SMG6	0.0383	5.05
A_23_P101272	NM_017908	ZNF446	0.0487	5.04
A_24_P75376	NM_015070	ZC3H13	0.0458	5.01
A_32_P216332	AL122040	AL122040	0.0494	4.99

A_24_P263443	ENST00000259550	ENST00000259550	0.0396	4.96
A_24_P301655	NM_001772	CD33	0.0427	4.95
A_24_P396557	NM_019083	CCDC76	0.0277	4.92
A_24_P860703	ENST00000360896	ENST00000360896	0.0287	4.90
A_23_P256190	NM_014826	CDC42BPA	0.0468	4.88
A_23_P39525	NM_024785	FLJ22746	0.0444	4.87
A_24_P325992	NM_002310	LIFR	0.0186	4.76
A_23_P156807	AK024077	ORC6L	0.0127	4.75
A_24_P89843	NM_032687	CYHR1	0.0338	4.73
A_23_P430051	AK022471	NGLY1	0.0270	4.73
A_24_P195714	A_24_P195714	A_24_P195714	0.0465	4.72
A_23_P203702	THC2372472	THC2372472	0.0278	4.69
A_23_P258321	NM_015969	MRPS17	0.0424	4.68
A_24_P360601	NM_017948	NOL8	0.0402	4.68
A_23_P346265	NM_024312	GNPTAB	0.0474	4.67
A_23_P29985	AK022953	C4orf23	0.0377	4.62
A_32_P43349	BC040875	LOC400456	0.0376	4.59
A_24_P278552	NM_005797	EVA1	0.0385	4.56
A_23_P201538	NM_002228	JUN	0.0120	4.51
A_24_P918875	THC2400529	THC2400529	0.0274	4.48
A_23_P392575	NM_080671	KCNE4	0.0310	4.47
A_23_P107507	NM_006807	CBX1	0.0460	4.47
A_23_P400603	NM_203448	RP11-262H14.4	0.0308	4.45
A_32_P137819	THC2385462	THC2385462	0.0215	4.41
A_24_P136441	AK023121	AK023121	0.0423	4.38
A_32_P159574	BC028022	BC028022	0.0435	4.38
A_23_P140277	NM_015180	SYNE2	0.0418	4.38
A_23_P255203	NM_033225	CSMD1	0.0487	4.37
A_24_P625683	A_24_P625683	A_24_P625683	0.0054	4.36
A_23_P208358	NM_000991	RPL28	0.0380	4.33
A_24_P237559	AK096804	LNPEP	0.0155	4.32
A_24_P935839	ENST00000333309	ENST00000333309	0.0488	4.30
A_23_P47991	NM_015335	THRAP2	0.0432	4.30
A_24_P942648	AL133642	EVL	0.0137	4.26
A_24_P221198	NM_203397	LOC255374	0.0373	4.24
A_23_P14928	NM_020312	COQ9	0.0497	4.23
A_32_P110086	A_32_P110086	A_32_P110086	0.0090	4.22
A_24_P218001	NM_021148	ZNF273	0.0322	4.21
A_24_P922909	AF272377	AF272377	0.0281	4.21
A_23_P64280	AY358681	AY358681	0.0470	4.19
A_23_P379159	NM_153252	BRWD3	0.0176	4.19
A_32_P52119	THC2250585	THC2250585	0.0233	4.19
A_24_P106542	NM_032784	RSPO3	0.0381	4.10
A_23_P313568	NM_181659	NCOA3	0.0160	4.10
A_24_P941167	NM_030641	APOL6	0.0334	4.09
A_23_P398491	NR_002208	MRPL42P5	0.0429	4.00

A_32_P728662	BC032246	ZNF44	0.0408	3.97
A_24_P249784	NM_178037	RAB6IP2	0.0131	3.90
A_23_P74001	NM_005621	S100A12	0.0274	3.85
A_32_P215143	XM_934182	LOC647022	0.0466	3.84
A_24_P324787	NM_015493	ANKRD25	0.0121	3.81
A_24_P856722	AI791206	AI791206	0.0208	3.79
A_24_P159181	NM_005707	PDCD7	0.0409	3.75
A_23_P97517	NM_018037	RALGPS2	0.0034	3.74
A_24_P26114	NM_005109	OXSR1	0.0312	3.72
A_23_P2814	NM_005905	SMAD9	0.0451	3.72
A_32_P65061	THC2283359	THC2283359	0.0285	3.68
A_23_P117416	NM_145685	BRF1	0.0435	3.66
A_32_P227657	THC2378933	THC2378933	0.0344	3.64
A_23_P215806	NM_024498	ZNF117	0.0482	3.63
A_24_P306561	NM_014972	TCF25	0.0353	3.62
A_23_P406131	NM_020422	TMEM159	0.0470	3.53
A_32_P220519	NM_014985	CEP152	0.0444	3.53
A_24_P136629	CR607522	CR607522	0.0227	3.51
A_24_P792130	THC2275025	THC2275025	0.0497	3.50
A_23_P500936	NM_021784	FOXA2	0.0157	3.49
A_24_P237757	AL136621	ZMYM2	0.0485	3.47
A_32_P110372	NM_021097	SLC8A1	0.0375	3.45
A_32_P153892	ENST00000298453	ENST00000298453	0.0376	3.45
A_24_P16541	NM_015383	NBPF14	0.0359	3.40
A_24_P84822	NM_001033523	GUSBL1	0.0407	3.40
A_23_P152570	THC2408471	THC2408471	0.0165	3.40
A_23_P391637	NM_020705	TBC1D24	0.0463	3.39
A_24_P852082	AK125829	AK125829	0.0487	3.38
A_32_P54289	THC2287647	THC2287647	0.0132	3.34
A_23_P13425	NM_004356	CD81	0.0272	3.30
A_23_P10605	A_23_P10605	A_23_P10605	0.0269	3.29
A_32_P209735	BC048193	LOC646326	0.0489	3.28
A_24_P549553	NM_003316	TTC3	0.0462	3.27
A_32_P187663	NM_173539	ZNF596	0.0415	3.25
A_23_P105651	NM_013377	PDZRN4	0.0406	3.14
A_23_P218079	NM_018976	SLC38A2	0.0344	3.13
A_24_P754185	DQ335469	KIAA1109	0.0279	3.11
A_23_P11564	NM_014774	KIAA0494	0.0360	3.10
A_32_P84388	NM_178439	GMCL1	0.0476	3.09
A_23_P158593	NM_000093	COL5A1	0.0205	3.08
A_24_P643776	THC2437143	THC2437143	0.0399	3.07
A_24_P666482	NM_001006607	LOC474170	0.0473	3.06
A_24_P367812	BC028099	LOC650392	0.0438	3.02
A_32_P83465	NM_183372	NBPF11	0.0136	2.99
A_24_P363615	NM_145808	MTPN	0.0283	2.97
A_23_P110598	NM_139281	WDR36	0.0358	2.96

A_23_P85543	NM_007212	RNF2	0.0235	2.96
A_23_P101013	NM_007267	TMC6	0.0260	2.93
A_24_P111009	NM_014649	SAFB2	0.0484	2.93
A_32_P171181	NM_173638	NBPF15	0.0140	2.91
A_23_P390206	ENST00000358917	ENST00000358917	0.0363	2.87
A_32_P203219	NM_198794	MAP4K5	0.0486	2.83
A_24_P216253	NM_014902	DLGAP4	0.0478	2.81
A_32_P163594	A_32_P163594	A_32_P163594	0.0367	2.78
A_23_P343843	NM_145039	MGC16385	0.0392	2.76
A_24_P101072	ENST00000369326	ENST00000369326	0.0201	2.74
A_24_P913956	AK022038	AK022038	0.0453	2.71
A_24_P31275	NM_001678	ATP1B2	0.0461	2.69
A_24_P53353	NM_001025300	RAB12	0.0377	2.68
A_23_P9255	NM_003177	SYK	0.0419	2.67
A_24_P131785	NM_003704	C4orf8	0.0358	2.66
A_24_P163113	NM_017548	CDV3	0.0418	2.64
A_24_P135061	NM_183372	NBPF11	0.0266	2.64
A_32_P69475	ENST00000379870	ENST00000379870	0.0315	2.62
A_24_P208897	NM_173827	COX18	0.0312	2.61
A_23_P120442	NM_181659	NCOA3	0.0199	2.60
A_32_P70519	THC2397883	THC2397883	0.0429	2.60
A_24_P205008	AK094466	TSEN54	0.0495	2.60
A_24_P592871	CB250445	CB250445	0.0439	2.57
A_24_P661593	AK023696	LOC401504	0.0326	2.53
A_23_P27677	NM_001571	IRF3	0.0240	2.51
A_23_P161644	ENST00000310106	ENST00000310106	0.0319	2.44
A_23_P147098	NM_145808	MTPN	0.0498	2.44
A_24_P555791	NM_004667	HERC2	0.0289	2.43
A_23_P210323	AB011154	CEP68	0.0334	2.43
A_23_P8123	THC2402058	THC2402058	0.0204	2.40
A_24_P345081	NM_032264	NBPF3	0.0344	2.39
A_24_P177631	AL832120	RP11-535K18.3	0.0368	2.38
A_24_P933514	AK094334	AK094334	0.0320	2.37
A_24_P205154	BC015370	BC015370	0.0403	2.33
A_23_P215275	BC080576	BC080576	0.0375	2.33
A_32_P51524	CR627362	CR627362	0.0498	2.31
A_32_P57140	BC067238	JMJD1C	0.0466	2.31
A_32_P36143	CR602569	CR602569	0.0487	2.30
A_23_P201939	NM_005167	PPM1J	0.0331	2.30
A_32_P144208	BC051298	LOC642826	0.0492	2.29
A_32_P220161	BI497361	BI497361	0.0177	2.25
A_32_P27698	NM_207366	FLJ44060	0.0390	2.20
A_23_P212749	NM_002111	HD	0.0428	2.17
A_23_P305292	AL137734	DKFZp586C0721	0.0390	2.16
A_32_P23360	NM_001005404	YPEL2	0.0249	2.16
A_23_P143178	NM_004139	LBP	0.0204	2.16

A_24_P102512	NM_012295	CABIN1	0.0178	2.13
A_23_P313223	NM_138471	LOC144097	0.0191	2.09
A_32_P99700	THC2415708	THC2415708	0.0319	2.07
A_23_P374104	NM_012098	ANGPTL2	0.0395	2.06
A_23_P168259	NM_025217	ULBP2	0.0295	2.06
A_23_P73348	NM_024335	IRX6	0.0459	2.04
A_24_P503710	AK000038	AK000038	0.0493	2.02
A_23_P9192	NM_005156	ROD1	0.0427	1.97
A_32_P74932	THC2379429	THC2379429	0.0421	1.93
A_24_P256325	NM_016316	REV1L	0.0366	1.93
A_23_P59988	NM_054028	AMAC1L2	0.0464	1.79
A_24_P2338	NM_022098	LOC63929	0.0476	1.74
A_32_P152696	A_32_P152696	A_32_P152696	0.0239	1.72
A_24_P192727	ENST00000224809	ENST00000224809	0.0491	1.64
A_32_P111235	BG612665	BG612665	0.0428	1.54
A_32_P60709	THC2406514	THC2406514	0.0329	1.47
A_23_P253734	NM_018941	CLN8	0.0006	1.32
A_24_P203964	NM_207331	LOC153561	0.0444	1.32
A_24_P51909	NM_006651	CPLX1	0.0421	1.21
A_32_P225209	THC2282972	THC2282972	0.0472	1.09
A_24_P288323	NM_139159	DPP9	0.0069	-1.05
A_24_P230282	NM_016378	VCX2	0.0444	-3.38
A_24_P359514	AF318367	C9orf86	0.0266	-3.99
A_23_P73809	NM_020871	LRCH2	0.0203	-3.99
A_23_P138105	NM_017638	MED18	0.0373	-7.85
A_24_P333136	NM_001004351	tcag7.1017	0.0090	-10.83

t12 Comparison				
**ProbeName**	**SystematicName**	**GeneName**	**p value**	**Fold (Very High / High)**
A_23_P1102	NM_001100	ACTA1	0.035	362.59
A_23_P49657	NM_005963	MYH1	0.024	271.21
A_23_P60742	NM_013292	MYLPF	0.004	190.61
A_24_P752862	THC2302027	THC2302027	0.034	8.42
A_23_P144326	NM_080874	ASB5	0.022	6.63
A_23_P15101	NM_024780	TMC5	0.038	5.22
A_24_P168581	NM_080425	GNAS	0.036	4.40
A_32_P44210	BC037328	BC037328	0.035	1.17
A_23_P82370	NM_024014	HOXA6	0.033	-0.58
A_24_P842172	AK090418	LOC349196	0.041	-1.11
A_23_P409287	NM_000458	TCF2	0.039	-1.11
A_23_P108734	NM_018436	ALLC	0.033	-1.20
A_23_P39746	A_23_P39746	A_23_P39746	0.042	-1.34
A_23_P165171	NM_017814	TMEM161A	0.037	-1.36
A_23_P3483	NM_021098	CACNA1H	0.015	-1.36
A_32_P150856	NR_002144	LOC407835	0.036	-1.36
A_24_P314688	NM_018218	USP40	0.039	-1.37
A_24_P911612	A_24_P911612	A_24_P911612	0.033	-1.37
A_23_P304524	NM_001040260	DCAMKL2	0.048	-1.38
A_23_P251196	A_23_P251196	A_23_P251196	0.048	-1.44
A_24_P66270	XM_934787	LOC653639	0.042	-1.47
A_23_P257057	NM_016647	C8orf55	0.046	-1.47
A_32_P507710	NM_199345	LOC375133	0.042	-1.48
A_24_P284783	NM_014337	PPIL2	0.028	-1.58
A_23_P25674	NM_001823	CKB	0.035	-1.59
A_24_P105501	NM_001428	ENO1	0.036	-1.59
A_24_P150803	NM_002773	PRSS8	0.035	-1.60
A_24_P148836	NM_173546	KLHDC8B	0.043	-1.61
A_23_P26117	NM_006715	MAN2C1	0.048	-1.63
A_23_P165028	NM_001348	DAPK3	0.049	-1.65
A_23_P56127	NM_019108	FLJ12886	0.048	-1.65
A_23_P49082	NM_012225	NUBP2	0.042	-1.68
A_24_P340390	NM_182485	CPEB2	0.031	-1.69
A_23_P392928	NM_178562	TSPAN33	0.029	-1.73
A_24_P317622	NM_031946	CENTG3	0.041	-1.78
A_24_P128683	NM_015680	C2orf24	0.031	-1.78
A_23_P27846	AB067468	KIAA1881	0.013	-1.79
A_24_P217330	NM_032806	C3orf39	0.049	-1.82
A_32_P165993	CB853344	CB853344	0.041	-1.82
A_23_P147383	NM_003801	GPAA1	0.018	-1.82
A_24_P373885	NM_152482	C19orf25	0.033	-1.87
A_23_P152245	NM_052987	CDK10	0.046	-1.89
A_24_P416059	NM_006715	MAN2C1	0.044	-1.90
A_23_P157333	NM_005232	EPHA1	0.049	-1.91

A_23_P203947	NM_030653	DDX11	0.039	-1.91
A_24_P41291	NM_198943	MGC52000	0.044	-1.92
A_23_P254254	NM_000199	SGSH	0.028	-1.93
A_23_P336708	NM_178502	DTX3	0.028	-1.93
A_24_P330822	NM_000458	TCF2	0.029	-1.94
A_23_P436336	NM_198284	LOC349114	0.046	-1.94
A_24_P66522	NM_014984	AZI1	0.043	-1.95
A_24_P366787	BC029785	FLJ42875	0.047	-1.95
A_23_P113417	NM_025161	C17orf70	0.050	-1.95
A_23_P330532	NM_080861	SPSB3	0.042	-1.96
A_24_P483871	A_24_P483871	A_24_P483871	0.028	-1.97
A_24_P6381	NM_178276	SERINC5	0.048	-1.97
A_24_P152144	A_24_P152144	A_24_P152144	0.034	-2.01
A_23_P30315	NM_033342	TRIM7	0.046	-2.01
A_23_P251898	NM_152743	C7orf27	0.049	-2.01
A_32_P130872	BQ441611	BQ441611	0.036	-2.02
A_23_P88909	NM_004209	SYNGR3	0.042	-2.04
A_23_P80136	NM_003683	D21S2056E	0.029	-2.06
A_24_P393372	AK098354	AK098354	0.034	-2.07
A_23_P39185	NM_138412	RDH13	0.050	-2.11
A_23_P49391	NM_016212	TP53TG3	0.029	-2.15
A_24_P13285	NM_006741	PPP1R1A	0.044	-2.15
A_23_P77980	NM_000342	SLC4A1	0.025	-2.16
A_24_P385336	NM_152696	HIPK1	0.036	-2.17
A_23_P62361	NM_014235	UBL4A	0.042	-2.20
A_23_P158533	NM_021008	DEAF1	0.030	-2.20
A_23_P74619	NM_003944	SELENBP1	0.017	-2.21
A_23_P259357	NM_001001479	SLC35E4	0.048	-2.21
A_24_P298360	NM_021070	LTBP3	0.029	-2.23
A_23_P15067	NM_138769	RHOT2	0.027	-2.24
A_23_P210581	NM_002237	KCNG1	0.030	-2.26
A_32_P7308	THC2407640	THC2407640	0.027	-2.28
A_23_P166491	NM_015705	RUTBC3	0.032	-2.28
A_23_P56163	NM_002850	PTPRS	0.043	-2.29
A_32_P102383	THC2323059	THC2323059	0.048	-2.29
A_23_P70127	NM_017510	TMED9	0.045	-2.34
A_23_P1641	NM_005133	RCE1	0.041	-2.34
A_23_P43296	NM_012162	FBXL6	0.022	-2.35
A_23_P87791	NM_001093	ACACB	0.040	-2.38
A_23_P300174	NM_173620	HEXDC	0.016	-2.38
A_23_P142835	NM_004082	DCTN1	0.045	-2.38
A_24_P145103	BC001335	BC001335	0.039	-2.40
A_23_P45976	NM_002885	RAP1GAP	0.022	-2.43
A_23_P164316	BC044941	LOC646038	0.022	-2.47
A_24_P322395	NM_080732	EGLN2	0.028	-2.48
A_24_P113815	NM_182838	SLC35E2	0.041	-2.49

A_23_P216996	NM_020145	SH3GLB2	0.043	-2.50
A_23_P304716	NM_019089	HES2	0.044	-2.50
A_23_P121665	NM_020777	SORCS2	0.042	-2.54
A_23_P66289	NM_024706	ZNF668	0.046	-2.56
A_23_P50857	NM_024527	ABHD8	0.046	-2.57
A_24_P375609	NM_001970	EIF5A	0.011	-2.59
A_23_P341349	NM_023083	CAPN10	0.034	-2.60
A_23_P339095	NM_178313	SPTBN1	0.041	-2.62
A_24_P2361	NM_004070	CLCNKA	0.021	-2.64
A_32_P107667	NM_001004351	tcag7.1017	0.043	-2.65
A_24_P108779	NM_024681	KCTD17	0.048	-2.66
A_23_P325498	NM_031856	PCDHA8	0.046	-2.70
A_32_P125803	BC034812	BC034812	0.011	-2.72
A_24_P356373	NM_032304	HAGHL	0.038	-2.73
A_24_P172304	NM_139028	ADAMTS13	0.012	-2.73
A_23_P39481	NM_019112	ABCA7	0.040	-2.75
A_32_P179615	THC2407386	THC2407386	0.040	-2.79
A_24_P15670	ENST00000316575	ENST00000316575	0.049	-2.80
A_24_P127174	ENST00000325371	ENST00000325371	0.036	-2.80
A_23_P371765	NM_032261	C21orf56	0.031	-2.82
A_23_P155979	NM_001963	EGF	0.030	-2.82
A_23_P62583	NM_001409	MEGF6	0.034	-2.82
A_32_P2362	NM_001001394	HCG3	0.044	-2.84
A_24_P32151	NM_182705	FAM101B	0.024	-2.84
A_32_P224234	AK123450	AK123450	0.002	-2.87
A_23_P108314	NM_030957	ADAMTS10	0.011	-2.89
A_23_P54576	NM_005550	KIFC3	0.040	-2.90
A_23_P346384	NM_176792	MRPL43	0.025	-2.91
A_23_P348063	NM_004711	SYNGR1	0.042	-2.96
A_24_P359405	NM_178502	DTX3	0.039	-2.97
A_32_P126485	BC047032	BC047032	0.030	-2.98
A_23_P211785	NM_003420	ZNF35	0.013	-2.98
A_24_P506816	NR_002158	OR2A20P	0.023	-3.00
A_23_P144704	ENST00000330731	ENST00000330731	0.028	-3.03
A_24_P203315	NM_182553	CNIH2	0.027	-3.04
A_24_P555619	THC2392444	THC2392444	0.043	-3.06
A_24_P381379	AK023831	FLJ13769	0.016	-3.10
A_23_P15751	NM_000160	GCGR	0.000	-3.15
A_32_P199901	ENST00000276173	ENST00000276173	0.020	-3.18
A_32_P124926	A_32_P124926	A_32_P124926	0.015	-3.18
A_32_P704982	BE379389	BE379389	0.014	-3.18
A_24_P886966	AK123483	AK123483	0.043	-3.21
A_23_P344000	NM_178537	B4GALNT4	0.038	-3.21
A_23_P8408	ENST00000223210	ENST00000223210	0.045	-3.23
A_23_P29079	NM_001002021	PFKL	0.037	-3.26
A_23_P122796	NM_016063	HDDC2	0.049	-3.38

A_24_P540057	NM_001739	CA5A	0.028	-3.38
A_23_P83818	NM_000093	COL5A1	0.050	-3.42
A_23_P339225	AK025264	KIAA1458	0.025	-3.44
A_23_P16047	NM_005282	GPR4	0.042	-3.54
A_32_P57989	BX115064	BX115064	0.037	-3.56
A_24_P93051	A_24_P93051	A_24_P93051	0.047	-3.56
A_23_P16476	NM_031918	KLF16	0.020	-3.57
A_23_P20697	NM_014694	ADAMTSL2	0.040	-3.57
A_23_P10995	NM_014483	RBMS3	0.030	-3.62
A_24_P127491	ENST00000330710	ENST00000330710	0.022	-3.66
A_24_P234105	NM_001029885	MGC10334	0.020	-3.70
A_24_P266466	NM_005293	GPR20	0.017	-3.76
A_23_P30582	NM_004174	SLC9A3	0.007	-3.78
A_23_P107775	NM_139172	MDAC1	0.036	-3.81
A_24_P641130	NM_080686	BAT2	0.034	-3.81
A_32_P122529	NM_001004351	tcag7.1017	0.041	-3.84
A_24_P255798	NM_030974	SHARPIN	0.029	-3.88
A_23_P312179	NM_015120	ALMS1	0.023	-3.90
A_23_P435833	NM_003934	FUBP3	0.043	-3.98
A_32_P84119	A_32_P84119	A_32_P84119	0.041	-4.00
A_24_P210637	NM_130468	D4ST1	0.022	-4.02
A_23_P321949	NM_000300	PLA2G2A	0.003	-4.03
A_23_P887	NM_014002	IKBKE	0.037	-4.03
A_24_P173727	NM_002617	PEX10	0.039	-4.12
A_24_P26496	NM_004557	NOTCH4	0.046	-4.13
A_24_P36890	NM_002885	RAP1GAP	0.010	-4.15
A_23_P146922	NM_000820	GAS6	0.033	-4.15
A_24_P912021	AK021785	AK021785	0.038	-4.15
A_24_P926820	AF159295	AF159295	0.038	-4.19
A_23_P77971	NM_000419	ITGA2B	0.040	-4.22
A_24_P388536	NM_025222	TMEM113	0.031	-4.25
A_24_P56689	NM_003456	ZNF205	0.035	-4.26
A_24_P61537	NM_001823	CKB	0.046	-4.28
A_24_P25640	J03651	J03651	0.019	-4.29
A_23_P22473	NM_012280	FTSJ1	0.029	-4.32
A_23_P166297	NM_207630	ABCG1	0.049	-4.33
A_23_P361482	NM_153712	TTL	0.045	-4.36
A_32_P44073	BQ333643	BQ333643	0.019	-4.39
A_23_P210297	A_23_P210297	A_23_P210297	0.043	-4.39
A_23_P391857	NM_004452	ESRRB	0.025	-4.39
A_23_P350295	ENST00000383620	ENST00000383620	0.019	-4.43
A_23_P18518	NM_152402	TRAM1L1	0.034	-4.43
A_32_P453321	NM_001004307	MGC33556	0.048	-4.48
A_24_P16326	NM_002942	ROBO2	0.043	-4.51
A_32_P187827	BC063633	LOC283970	0.033	-4.55
A_23_P78903	NM_024877	CNTD2	0.040	-4.58

A_23_P211196	NM_058188	C21orf67	0.002	-4.67
A_23_P37785	ENST00000304372	ENST00000304372	0.040	-4.71
A_24_P52882	NM_003806	HRK	0.042	-4.72
A_23_P330537	NM_080861	SPSB3	0.025	-4.78
A_24_P295633	NM_024841	FLJ14213	0.047	-4.78
A_24_P916972	AK023928	AK023928	0.024	-4.79
A_23_P136623	NM_183357	ADCY5	0.025	-4.79
A_32_P92840	AI830766	AI830766	0.035	-4.89
A_24_P103327	NM_004472	FOXD1	0.047	-4.89
A_24_P370626	NM_001004285	DFFB	0.041	-4.93
A_24_P151305	NM_016270	KLF2	0.039	-4.94
A_24_P205252	NM_001001971	FAM13C1	0.036	-4.99
A_24_P280465	BC028606	NY-REN-7	0.046	-5.01
A_32_P229132	NM_020066	FMN2	0.002	-5.01
A_23_P364355	NM_001040067	LOC647353	0.027	-5.01
A_32_P91743	THC2332864	THC2332864	0.016	-5.02
A_23_P103971	NM_178134	CYP4Z1	0.015	-5.07
A_23_P309281	NM_005543	INSL3	0.042	-5.08
A_24_P251040	NM_057091	ARTN	0.045	-5.08
A_24_P565898	A_24_P565898	A_24_P565898	0.047	-5.14
A_23_P153183	AB020709	CNKSR2	0.021	-5.15
A_24_P30257	NM_002478	MYOD1	0.031	-5.22
A_23_P162719	NM_030932	DIAPH3	0.024	-5.29
A_23_P16089	NM_004230	EDG5	0.001	-5.31
A_24_P31421	NM_015711	GLTSCR1	0.039	-5.34
A_24_P5550	NM_024297	PHF23	0.029	-5.35
A_23_P151970	NM_015322	FEM1B	0.025	-5.38
A_23_P201156	NM_021189	IGSF4B	0.037	-5.38
A_24_P281228	AL050020	DKFZP564C196	0.045	-5.42
A_24_P924752	M14087	M14087	0.028	-5.44
A_24_P879135	BQ015140	BQ015140	0.021	-5.45
A_32_P207986	A_32_P207986	A_32_P207986	0.047	-5.45
A_24_P831309	NM_207401	FLJ45717	0.020	-5.48
A_24_P911928	ENST00000331406	ENST00000331406	0.007	-5.49
A_24_P289504	A_24_P289504	A_24_P289504	0.046	-5.53
A_23_P111506	ENST00000342773	ENST00000342773	0.024	-5.59
A_23_P338113	NP101106	NP101106	0.017	-5.59
A_24_P90900	NM_001907	CTRL	0.048	-5.60
A_24_P316454	BC022826	BC022826	0.045	-5.65
A_24_P181178	NM_178040	RAB6IP2	0.008	-5.66
A_23_P11902	A_23_P11902	A_23_P11902	0.020	-5.66
A_23_P119222	NM_020415	RETN	0.035	-5.68
A_23_P398854	NM_173660	C4orf25	0.032	-5.69
A_24_P920938	THC2315706	THC2315706	0.047	-5.69
A_23_P314024	NM_018950	HLA-F	0.031	-5.88
A_24_P127719	NM_201589	MAFA	0.044	-5.92

A_24_P40775	ENST00000248668	ENST00000248668	0.030	-5.97
A_24_P207195	NM_024336	IRX3	0.008	-6.01
A_24_P307384	A_24_P307384	A_24_P307384	0.042	-6.02
A_23_P28139	NM_002980	SCTR	0.024	-6.04
A_24_P767972	A_24_P767972	A_24_P767972	0.016	-6.05
A_32_P117127	ENST00000330336	ENST00000330336	0.023	-6.08
A_24_P564462	CN430223	CN430223	0.002	-6.08
A_24_P368748	A_24_P368748	A_24_P368748	0.049	-6.08
A_32_P144634	THC2348391	THC2348391	0.028	-6.15
A_24_P918137	AK096487	AK096487	0.027	-6.16
A_32_P35375	NM_021008	DEAF1	0.035	-6.18
A_32_P111394	THC2441040	THC2441040	0.045	-6.23
A_24_P44916	NM_145057	CDC42EP5	0.020	-6.28
A_24_P912720	X89741	CHRNA4	0.041	-6.28
A_24_P125977	NM_033184	KRTAP2-4	0.047	-6.30
A_23_P257014	NM_005873	RGS19	0.017	-6.32
A_23_P147782	NM_031309	SCRT1	0.018	-6.33
A_24_P208513	NM_006522	WNT6	0.038	-6.37
A_24_P551530	A_24_P551530	A_24_P551530	0.042	-6.39
A_23_P141005	NM_001144	AMFR	0.034	-6.42
A_23_P407601	AJ307469	C8orf6	0.019	-6.47
A_24_P5750	NM_005551	KLK2	0.004	-6.48
A_24_P208774	NM_153284	HYAL1	0.037	-6.49
A_24_P384397	NM_133452	RAVER1	0.027	-6.52
A_32_P32338	THC2256652	THC2256652	0.022	-6.52
A_24_P10890	NM_015366	PRR5	0.029	-6.54
A_24_P218074	NM_207336	ZNF467	0.033	-6.63
A_23_P84063	NM_016522	HNT	0.037	-6.65
A_32_P150928	NM_012262	HS2ST1	0.026	-6.66
A_32_P187160	A_32_P187160	A_32_P187160	0.029	-6.72
A_23_P375419	NM_144965	TTC16	0.011	-6.74
A_23_P308464	AK055181	ARID2	0.032	-6.74
A_23_P92275	NM_153002	GPR156	0.045	-6.81
A_23_P348524	NM_001007125	LOC198437	0.040	-6.83
A_32_P86180	ENST00000369123	ENST00000369123	0.020	-6.87
A_32_P9368	BX375060	BX375060	0.040	-6.93
A_24_P7873	ENST00000333066	ENST00000333066	0.043	-6.93
A_23_P86493	NM_006562	LBX1	0.023	-6.94
A_24_P215445	NM_005169	PHOX2A	0.031	-6.94
A_24_P39484	AK025430	AK025430	0.045	-6.98
A_24_P503729	AK021531	AK021531	0.038	-6.98
A_23_P401626	NM_174919	LOC201175	0.020	-7.11
A_24_P927245	A_24_P927245	A_24_P927245	0.016	-7.12
A_24_P237231	NM_173354	SNF1LK	0.034	-7.18
A_32_P222450	NM_015444	TMEM158	0.027	-7.21
A_23_P200267	AB007895	PCNXL2	0.032	-7.23

A_24_P213788	BC020624	LOC641518	0.018	-7.25
A_24_P929946	NP111687	NP111687	0.007	-7.30
A_24_P757154	THC2375008	THC2375008	0.028	-7.32
A_23_P3413	NM_130901	OTUD7A	0.033	-7.33
A_32_P50264	BF513091	BF513091	0.016	-7.35
A_24_P253293	NM_014360	NKX2-8	0.025	-7.35
A_24_P745960	A_24_P745960	A_24_P745960	0.031	-7.36
A_24_P212826	AK098804	ZNF525	0.042	-7.50
A_23_P149707	ENST00000235180	ENST00000235180	0.038	-7.50
A_24_P845649	A_24_P845649	A_24_P845649	0.024	-7.51
A_32_P35759	BX118285	BX118285	0.030	-7.51
A_23_P346357	NM_173832	ZFP41	0.003	-7.54
A_23_P63083	NM_018489	ASH1L	0.012	-7.57
A_24_P14731	NM_013271	PCSK1N	0.015	-7.59
A_32_P196247	THC2444634	THC2444634	0.040	-7.64
A_32_P233211	A_32_P233211	A_32_P233211	0.030	-7.68
A_23_P96072	NM_021569	GRIN1	0.014	-7.73
A_24_P273063	NM_024812	BAALC	0.029	-7.74
A_32_P230059	A_32_P230059	A_32_P230059	0.025	-7.79
A_23_P54770	NM_018690	APOB48R	0.047	-7.80
A_23_P132277	NM_006739	MCM5	0.015	-7.85
A_32_P216602	THC2306788	THC2306788	0.037	-7.85
A_24_P73158	NM_004111	FEN1	0.000	-7.94
A_24_P919340	A_24_P919340	A_24_P919340	0.046	-7.97
A_23_P11729	NM_015872	ZBTB7B	0.000	-7.97
A_23_P11005	NM_014272	ADAMTS7	0.031	-8.03
A_24_P780709	A_24_P780709	A_24_P780709	0.012	-8.16
A_23_P364592	NM_020742	NLGN4X	0.002	-8.17
A_23_P256663	NM_003614	GALR3	0.035	-8.29
A_24_P273378	NM_172215	CAMKK2	0.011	-8.32
A_32_P83997	NM_201430	RTN3	0.022	-8.36
A_32_P70341	THC2348879	THC2348879	0.049	-8.40
A_24_P57898	NM_080606	BHLHB4	0.042	-8.40
A_32_P88317	ENST00000302096	ENST00000302096	0.035	-8.46
A_23_P26294	NM_012467	TPSG1	0.033	-8.48
A_32_P132883	THC2282416	THC2282416	0.008	-8.51
A_24_P102406	BC014871	BC014871	0.049	-8.57
A_24_P144465	NM_022107	GPSM3	0.030	-8.59
A_24_P938313	NM_002012	FHIT	0.019	-8.65
A_24_P12651	NM_171982	TRIM35	0.048	-8.72
A_32_P171984	A_32_P171984	A_32_P171984	0.008	-8.77
A_24_P115967	NM_006133	C11orf11	0.024	-8.81
A_24_P49199	NM_181789	GLDN	0.035	-8.86
A_23_P500824	NM_133499	SYN1	0.022	-8.96
A_32_P78395	THC2374967	THC2374967	0.037	-8.97
A_24_P791968	THC2445319	THC2445319	0.039	-8.99

A_24_P112803	NM_005986	SOX1	0.027	-9.07
A_24_P173294	NM_031918	KLF16	0.020	-9.07
A_24_P293089	NM_152600	ZNF579	0.031	-9.09
A_24_P345837	NM_002448	MSX1	0.031	-9.11
A_23_P342612	NM_001194	HCN2	0.023	-9.18
A_23_P104413	NM_033178	DUX4	0.011	-9.18
A_23_P6818	NM_020163	SEMA3G	0.017	-9.20
A_32_P132422	A_32_P132422	A_32_P132422	0.011	-9.22
A_23_P24507	AL833655	AL833655	0.039	-9.27
A_23_P150162	NM_000797	DRD4	0.011	-9.31
A_24_P216165	NM_004364	CEBPA	0.045	-9.45
A_24_P110101	ENST00000327579	ENST00000327579	0.048	-9.49
A_32_P160200	THC2342129	THC2342129	0.012	-9.63
A_24_P728006	XM_933130	LOC653316	0.014	-9.65
A_23_P310439	NM_144727	CRYGN	0.010	-9.67
A_24_P727884	U10991	C11orf41	0.044	-9.84
A_24_P90923	ENST00000321214	ENST00000321214	0.037	-9.90
A_24_P235098	NM_033260	FOXQ1	0.015	-9.94
A_23_P201747	ENST00000375486	ENST00000375486	0.032	-9.94
A_24_P303874	BC034752	C9orf62	0.020	-9.99
A_23_P206466	NM_020764	CASKIN1	0.021	-10.00
A_24_P315500	AK094323	AK094323	0.016	-10.03
A_23_P59976	NM_002066	GML	0.044	-10.03
A_23_P54573	NM_153813	ZFPM1	0.030	-10.05
A_24_P917363	A_24_P917363	A_24_P917363	0.009	-10.08
A_23_P107394	NM_032580	HES7	0.025	-10.13
A_23_P125686	NR_002226	INGX	0.048	-10.17
A_32_P231880	A_32_P231880	A_32_P231880	0.012	-10.24
A_23_P81153	NM_033632	FBXW7	0.027	-10.43
A_24_P333364	NM_001042535	CENTG3	0.031	-10.44
A_32_P61074	A_32_P61074	A_32_P61074	0.031	-10.51
A_32_P210390	AA991174	AA991174	0.021	-10.60
A_32_P233278	THC2438327	THC2438327	0.029	-10.61
A_24_P204269	NM_170606	MLL3	0.016	-10.65
A_24_P203953	BC037281	LOC439951	0.019	-10.69
A_23_P127525	NM_005238	ETS1	0.013	-10.72
A_24_P24856	BC080624	BC080624	0.012	-10.76
A_24_P20807	NM_030567	PRR7	0.025	-10.77
A_23_P84762	NM_032789	PARP10	0.024	-10.80
A_23_P153549	NM_000836	GRIN2D	0.021	-10.90
A_24_P280497	AF490258	KIAA1545	0.030	-10.93
A_24_P324479	AK024930	AK024930	0.036	-10.94
A_32_P170368	AI878825	AI878825	0.012	-11.02
A_24_P108401	AF335478	KLK3	0.031	-11.03
A_23_P35534	NM_020999	NEUROG3	0.022	-11.07
A_23_P346309	NM_138763	BAX	0.004	-11.14

A_24_P475864	BC042920	RRN3	0.042	-11.14
A_23_P304530	NM_178438	LCE5A	0.013	-11.32
A_32_P188143	THC2375818	THC2375818	0.045	-11.33
A_24_P48522	NM_018111	FLJ10490	0.015	-11.37
A_24_P307306	A_24_P307306	A_24_P307306	0.014	-11.50
A_24_P376451	ENST00000381826	ENST00000381826	0.028	-11.52
A_23_P375524	NM_178352	LCE1D	0.024	-11.62
A_23_P15135	NM_032805	ZNF206	0.035	-11.67
A_32_P234913	A_32_P234913	A_32_P234913	0.014	-11.76
A_24_P102456	BC028232	BC028232	0.025	-11.88
A_24_P247169	A_24_P247169	A_24_P247169	0.026	-11.90
A_32_P181183	AI918632	AI918632	0.018	-11.91
A_32_P101073	A_32_P101073	A_32_P101073	0.025	-11.98
A_24_P780393	A_24_P780393	A_24_P780393	0.019	-12.07
A_23_P372368	NM_001040140	C21orf87	0.012	-12.16
A_23_P21057	NM_052838	1-Sep	0.030	-12.23
A_23_P150664	NM_001025295	IFITM5	0.033	-12.28
A_24_P416595	NM_174945	ZNF575	0.014	-12.33
A_32_P90455	ENST00000376615	ENST00000376615	0.012	-12.41
A_24_P714316	A_24_P714316	A_24_P714316	0.014	-12.65
A_23_P25790	NM_022478	CDH24	0.014	-12.73
A_24_P399871	NM_021096	CACNA1I	0.008	-12.82
A_23_P50773	NM_025021	CRTC1	0.005	-12.88
A_23_P115118	NM_181809	BMP8A	0.008	-12.92
A_32_P20288	BC063316	FBXL17	0.016	-13.06
A_24_P246636	XM_927199	LOC643940	0.035	-13.09
A_23_P79066	NM_020172	SPPL2B	0.027	-13.10
A_32_P350	CA503034	CA503034	0.013	-13.32
A_24_P38572	NM_130793	NOL6	0.042	-13.34
A_32_P160254	XM_929673	DKFZP434I0714	0.014	-13.61
A_24_P923765	NM_203304	RKHD1	0.017	-13.75
A_24_P147540	AK096643	FLJ22184	0.019	-13.76
A_24_P417922	A_24_P417922	A_24_P417922	0.011	-13.80
A_24_P6933	AL137479	TPCN2	0.013	-14.00
A_32_P85711	A_32_P85711	A_32_P85711	0.016	-14.04
A_24_P285158	BC067080	FCRLM2	0.022	-14.20
A_23_P146885	NM_018949	UTS2R	0.014	-14.51
A_32_P112100	A_32_P112100	A_32_P112100	0.008	-14.88
A_32_P140312	A_32_P140312	A_32_P140312	0.009	-15.00
A_32_P151317	BI818647	BI818647	0.013	-15.00
A_24_P7785	A_24_P7785	A_24_P7785	0.016	-15.37
A_23_P140190	ENST00000360329	ENST00000360329	0.046	-15.42
A_24_P113725	NM_005634	SOX3	0.019	-15.49
A_24_P279060	NM_005477	HCN4	0.006	-15.96
A_32_P180538	AI354226	AI354226	0.045	-16.19
A_32_P52713	THC2279765	THC2279765	0.017	-16.23

A_24_P15640	NM_001014373	C19orf31	0.010	-16.26
A_24_P914134	ENST00000379440	ENST00000379440	0.005	-16.29
A_32_P71864	A_32_P71864	A_32_P71864	0.025	-16.35
A_24_P401294	NM_207453	FLJ35934	0.046	-16.35
A_32_P226918	NM_006256	PKN2	0.011	-16.53
A_23_P164927	NM_012451	SYNGR4	0.024	-16.66
A_24_P270044	NM_024769	ASAM	0.022	-16.74
A_24_P42107	A_24_P42107	A_24_P42107	0.009	-16.77
A_32_P65793	A_32_P65793	A_32_P65793	0.014	-16.77
A_24_P461664	A_24_P461664	A_24_P461664	0.013	-16.86
A_23_P333129	XM_495854	LOC440013	0.006	-16.97
A_24_P533990	A_24_P533990	A_24_P533990	0.009	-17.07
A_24_P762669	XM_376567	KIAA1856	0.011	-17.14
A_32_P15887	A_32_P15887	A_32_P15887	0.004	-17.17
A_24_P349788	ENST00000325814	ENST00000325814	0.028	-17.25
A_24_P37665	NM_022042	SLC26A1	0.019	-17.42
A_24_P37887	NM_199243	GPR150	0.008	-17.47
A_23_P339744	NR_002797	LOC255783	0.001	-17.69
A_23_P97652	NM_207370	GPR153	0.009	-17.70
A_24_P641742	A_24_P641742	A_24_P641742	0.029	-17.89
A_24_P62367	NM_004746	DLGAP1	0.034	-17.99
A_23_P21570	NM_173159	NPAS3	0.012	-18.82
A_23_P141699	NM_032510	PARD6G	0.006	-19.70
A_24_P348083	BC093850	C18orf23	0.007	-23.27
A_23_P100263	NM_198390	CMIP	0.015	-26.22
A_23_P343900	ENST00000327436	ENST00000327436	0.049	-26.31
A_23_P69652	NM_080819	GPR78	0.014	-27.31
A_23_P34554	NM_000721	CACNA1E	0.031	-27.87
A_24_P10674	NM_052948	SNX26	0.002	-48.73
A_23_P354146	NM_005546	ITK	0.013	-62.08
A_23_P31725	NM_001715	BLK	0.012	-64.78
A_32_P203959	BM547196	BM547196	0.001	-75.83
