# Supplementary material for: Intragraft transcriptional profiling of renal transplant patients with tubular dysfunction reveals mechanisms underlying graft injury and recovery
Source: Hum Genomics. 2016 Jan 7;10:2. doi: 10.1186/s40246-015-0059-6 (PMC4705764; doi:10.1186/s40246-015-0059-6)
Supplement: Additional file 2: — High-hub, hub, and bottleneck genes identified in each comparison. Hubs were defined as highly connected nodes according to node degree values. High-hubs are top-ranked hubs presenting also high betweenness centrality values. Bottleneck genes were classified as nodes with high betweenness centrality but low node degree values. [file 40246_2015_59_MOESM2_ESM.pdf]

GROUP 1 COMPARISON		
**Nodes**	**Gene**	**Type**
**High-hubs**	Adnp	Co-expressed
	Birc2	Co-expressed
	Btaf1	Co-expressed
	Clk1	Co-expressed
	Dmtf1	Co-expressed
	Dmxl1	Co-expressed
	KIAA0528	Co-expressed
	Luc7l3	Co-expressed
	Nup153	Co-expressed
	Pja2	Co-expressed
	Sacm1l	Co-expressed
	Thoc1	Co-expressed
	Satb1	Co-expressed
	Scaf8	Co-expressed
	Sec24b	Co-expressed
	Serinc1	Co-expressed
	Shoc2	Co-expressed
	Slc35a1	Co-expressed
	Smndc1	Co-expressed
	Tmem123	Co-expressed
	Top2b	Co-expressed
	Trappc8	Co-expressed
	Tsc1	Differentially expressed
	Ncl	Differentially expressed
	Mtr	Co-expressed
	Thoc1	Co-expressed
	Zcchc11	Co-expressed
	Stam	Co-expressed
	Tspyl4	Co-expressed
	Ankrd17	Co-expressed
	Scp2	Co-expressed

Nodes	Gene	Type
	Agbl2	Differentially expressed
	Ifna4	Differentially expressed
	L1cam	Differentially expressed
	Fut6	Differentially expressed
	Bai3	Differentially expressed
	Tnpo2	Differentially expressed
	Fxyd2	Differentially expressed
	Ankrd43	Differentially expressed
	Tyk2	Differentially expressed
	Krt33a	Differentially expressed
	Pacsin2	Differentially expressed

Bottlenecks	Itgal	Differentially expressed
	Atp1b1	Differentially expressed
	Znf516	Differentially expressed
	Adar	Differentially expressed
	Agap4	Differentially expressed
	Kctd20	Differentially expressed
	Epb49	Differentially expressed
	Zfp14	Differentially expressed
	Camta2	Differentially expressed
	Cytip	Differentially expressed
	Bach2	Differentially expressed
	Rad51ap1	Differentially expressed
	Zkscan1	Co-expressed
	Mtmr4	Co-expressed
	Wnt10b	Differentially expressed
	Pom121	Differentially expressed

Nodes	Gene	Type
Hubs	Ttc37	Co-expressed
	Bmi1	Differentially expressed
	Pcnp	Co-expressed
	Srsf11	Co-expressed
	Upf2	Co-expressed
	Dusp11	Co-expressed
	Pitpnb	Co-expressed
	Rab11fip2	Co-expressed
	Rwdd3	Co-expressed
	Ranbp6	Co-expressed
	Haus3	Co-expressed
	Nek7	Co-expressed
	Uba3	Co-expressed
	March7	Co-expressed
	Bnip2	Co-expressed
	Matr3	Co-expressed
	Vps4b	Differentially expressed
	Prpf39	Differentially expressed
	G3bp2	Co-expressed
	Sep15	Co-expressed
	Zzz3	Co-expressed
	Chd1	Co-expressed

GROUP 2 COMPARISON		
**Nodes**	**Gene**	**Type**
**High-hubs**	C17orf62	Differentially expressed
	C1r	Differentially expressed
	CD3E	Differentially expressed
	HLA-E	Differentially expressed
	Il10ra	Differentially expressed
	Mgat1	Differentially expressed
	Ptpn6	Differentially expressed
	Rgs19	Differentially expressed
	Rhog	Differentially expressed
	Rps6ka1	Differentially expressed
	Serping1	Differentially expressed
	Arrb2	Co-expressed
	Coro1a	Co-expressed
	Csf1r	Co-expressed
	Emp3	Co-expressed
	Gmfg	Co-expressed
	Hcls1	Co-expressed
	Plekho2	Co-expressed
	Ptprcap	Co-expressed
	Sash3	Co-expressed
	Sipa1	Co-expressed
	Tcirg1	Co-expressed

Nodes	Gene	Type
**Bottlenecks**	Pkm2	Differentially expressed
	Mmp12	Differentially expressed
	Tuba1a	Differentially expressed
	Rtn1	Differentially expressed
	Nme4	Differentially expressed
	Olfml1	Differentially expressed
	Cdc25b	Differentially expressed
	Map7d1	Differentially expressed
	Fxyd6	Differentially expressed
	Twf2	Differentially expressed
	Igfbp4	Co-expressed
	Limd2	Differentially expressed
	Gsdmd	Differentially expressed
	Fmod	Differentially expressed
	Lect1	Differentially expressed
	Ptms	Differentially expressed
	Cyp4f12	Differentially expressed
	Plekha4	Differentially expressed
	Plcb3	Differentially expressed
	Gipc1	Differentially expressed

	Agpat2	Differentially expressed
	Akt1	Differentially expressed
	Cpz	Differentially expressed
	Slc5a5	Differentially expressed
	Lsr	Differentially expressed
	Ogt	Differentially expressed

**Nodes**	**Gene**	**Type**
**Hubs**	C1orf38	Differentially expressed
	Tyrobp	Co-expressed
	Spi1	Co-expressed
	Slc15a3	Co-expressed
	Plekho1	Co-expressed
	Nckap1l	Co-expressed
	Ncf4	Differentially expressed
	Ncf2	Differentially expressed
	Myo1f	Differentially expressed
	Man2b1	Co-expressed
	Lst1	Co-expressed
	Lsp1	Differentially expressed
	Lilrb3	Co-expressed
	Lcp2	Co-expressed
	Lcp1	Co-expressed
	Laptm5	Differentially expressed
	Itgb2	Co-expressed
	Itgal	Differentially expressed
	Ifi30	Co-expressed
	HLA-DPB1	Co-expressed
	HLA-DMB	Co-expressed
	Hck	Co-expressed
	Gpsm3	Co-expressed
	Fcer1g	Co-expressed
	CD53	Co-expressed
	CD14	Co-expressed
	C1qb	Co-expressed

T0 COMPARISON		
**Nodes**	**Gene**	**Type**
**High-hubs**	Ankrd36b	Co-expressed
	Satb1	Co-expressed
	Trappc8	Co-expressed
	Mycbp2	Co-expressed
	Baz2b	Co-expressed
	Znf136	Co-expressed
	Top2b	Co-expressed
	Btaf1	Co-expressed
	Fnbp4	Co-expressed
	Dmtf1	Co-expressed
	Prr11	Co-expressed
	Ercc5	Co-expressed
	Clk1	Co-expressed
	Nxf1	Co-expressed
	Chd8	Co-expressed
	Ppip5k2	Co-expressed
	Pja2	Co-expressed

Nodes	Gene	Type
**Bottlenecks**	Agrp	Differentially expressed
	Lbp	Differentially expressed
	Cdv3	Differentially expressed
	Tmem159	Differentially expressed
	Syk	Differentially expressed
	Dlgap4	Differentially expressed
	Cep152	Differentially expressed
	Fam129a	Differentially expressed
	Cabin1	Differentially expressed
	Slc38a2	Differentially expressed
	KIAA0494	Differentially expressed
	Fancg	Differentially expressed
	Edc4	Co-expressed
	Evl	Differentially expressed
	Haus2	Co-expressed
	Madd	Co-expressed
	Tcf4	Co-expressed
	Rbm10	Co-expressed
	Supt5h	Co-expressed
	Sympk	Differentially expressed
	Arvcf	Differentially expressed
	Cd81	Differentially expressed
	Arhgap17	Differentially expressed

Nodes	Gene	Type

**Hubs**	Luc7l3	Co-expressed
	Arglu1	Co-expressed
	Tcerg1	Co-expressed
	Zcchc11	Co-expressed
	Mtf2	Co-expressed
	Nol8	Differentially expressed
	Pum1	Co-expressed
	Ankrd17	Differentially expressed
	Ttc3	Co-expressed
	Haus3	Co-expressed
	Smg1	Co-expressed
	Rbm6	Co-expressed
	Nup153	Co-expressed
	Pcmtd2	Co-expressed
	Nipbl	Co-expressed
	Tia1	Co-expressed
	KIAA0528	Co-expressed
	Srsf11	Co-expressed

T12 COMPARISON		
**Nodes**	**Gene**	**Type**
**High-hubs**	AC010336.1	Differentially expressed
	Aldoa	Co-expressed
	Avp	Co-expressed
	B4galt2	Co-expressed
	Ccdc9	Co-expressed
	Clc	Co-expressed
	Cox6a2	Co-expressed
	Crybb3	Co-expressed
	Gfra4	Co-expressed
	Irf3	Co-expressed
	Lmf2	Co-expressed
	Map3k10	Co-expressed
	Med22	Co-expressed
	Mmp17	Co-expressed
	Myod1	Differentially expressed
	Pcsk1n	Differentially expressed
	Pip5k1c	Co-expressed
	Pnpla6	Co-expressed
	Rhbdl1	Co-expressed
	Slc4a3	Co-expressed
	Znf205	Differentially expressed

Nodes	Gene	Type
**Bottlenecks**	Hipk1	Differentially expressed
	Rtn3	Differentially expressed
	Syngr3	Differentially expressed
	Sema3g	Differentially expressed
	Apobr	Differentially expressed
	Ckb	Differentially expressed
	Sept1	Differentially expressed
	Col5a1	Differentially expressed
	Ash1l	Differentially expressed
	Pkn2	Differentially expressed
	Fhit	Differentially expressed
	Gpr4	Differentially expressed
	Rgs19	Differentially expressed
	Arfgap2	Differentially expressed
	Egln2	Differentially expressed
	Znf575	Differentially expressed
	Sptbn1	Differentially expressed
	Hyal1	Differentially expressed
	Rrn3	Differentially expressed

Nodes	Gene	Type

**Hubs**	Lrch4	Co-expressed
	Lhx1	Co-expressed
	Neurog1	Co-expressed
	Gpr153	Differentially expressed
	Tpsg1	Differentially expressed
	Phox2a	Differentially expressed
	Lbx1	Differentially expressed
	Hes7	Differentially expressed
	Hcn2	Differentially expressed
	Mast1	Co-expressed
	Znf579	Differentially expressed
	Dlgap3	Co-expressed
	Gpr150	Differentially expressed
	Ifitm5	Differentially expressed
	Fam193b	Co-expressed
	Sox18	Co-expressed
	Nkx2-8	Differentially expressed
	Foxc2	Co-expressed
	Cacna1e	Differentially expressed
	Neurog3	Differentially expressed
	Syngr4	Differentially expressed
	Lrp3	Co-expressed
	Grin2d	Differentially expressed
	Cadm3	Co-expressed
	Artn	Differentially expressed
	En2	Co-expressed
	Evx1	Co-expressed
	Foxb1	Co-expressed
	Galr3	Co-expressed
